# Supplementary material for: Relative Survival, Conditional Survival, and Causes of Death in Patients with Early Gastric Cancer, with a Focus on Differences Between Cardia and Non-Cardia Cancer
Source: Cancers (Basel). 2024 Dec 21;16(24):4262. doi: 10.3390/cancers16244262 (PMC11674421; doi:10.3390/cancers16244262)
Supplement: Supplementary file 1 [file cancers-16-04262-s001.zip › cancers-3327131-supplementary.pdf]

Causes	<1 years		1-2 years		2-5 years		>5 years		Total		Total	
	Observed (n)	SMR (95%CI)	Observed (n)	SMR (95%CI)	Observed (n)	Observed (n)	SMR (95%CI)	Observed (n)	SMR (95%CI)	Observed (n)	Patient	Mean Age at Event
Non-GC	4	536.36^{p}(146.14-1373.3)	1	137.71^{p}(3.49-767.27)	2	113.16^{p}(13.7-408.77)	1	19.81(0.5-110.37)	8	96.53^{p}(41.68-190.21)	53	38.29
GC	4	13,705.27^{p}(3734.23-35090.94)	2	7,395.38^{p}(895.61-26714.64)	2	2,898.88^{p}(351.07-10471.75)	1	594.02^{p}(15.04-3309.68)	9	3,065.75^{p}(1401.86-5819.75)	53	38.56
All Causes of Death	8	115.09^{p}(49.69-226.78)	3	48.93^{p}(10.09-142.99)	5	35.27^{p}(11.45-82.31)	3	9.81^{p}(2.02-28.68)	19	32.86^{p}(19.78-51.31)	53	38.76
Non-cancer causes	0	0(0-59.73)	0	0(0-68.59)	1	8.1(0.21-45.15)	1	3.94(0.1-21.98)	2	4.06(0.49-14.67)	53	41.5
Septicemia	0	0(0-6411.47)	0	0(0-7085.61)	0	0(0-2969.3)	0	0(0-1112.78)	0	0(0-652.52)	53	
Other Infectious and Parasitic Diseases including HIV	0	0(0-2251.26)	0	0(0-2982.14)	0	0(0-1379.51)	0	0(0-700.57)	0	0(0-341.08)	53	
Diabetes Mellitus	0	0(0-2755)	0	0(0-2948.93)	0	0(0-1194.66)	0	0(0-401.13)	0	0(0-248.01)	53	
Alzheimer's (ICD-9 and 10 only)	0	0(0-1160329.45)	0	0(0-810925.34)	0	0(0-236609.38)	0	0(0-41160.12)	0	0(0-32661.89)	53	
Cardiovascular Diseases	0	0(0-461.53)	0	0(0-496.1)	0	0(0-197.59)	0	0(0-67.21)	0	0(0-41.46)	53	
Cerebrovascular Diseases	0	0(0-3220.12)	0	0(0-3409.67)	0	0(0-1394.04)	0	0(0-525.19)	0	0(0-310.05)	53	
Pneumonia and Influenza	0	0(0-5372.64)	0	0(0-5587.79)	0	0(0-2511.68)	0	0(0-1044.96)	0	0(0-581.33)	53	
Chronic Obstructive Pulmonary Disease and Allied Cond	0	0(0-8333.16)	0	0(0-8149.88)	0	0(0-3322.28)	0	0(0-888.08)	0	0(0-598.9)	53	
Chronic Liver Disease and Cirrhosis	0	0(0-1954.62)	0	0(0-1956.8)	0	0(0-727.14)	0	0(0-234.69)	0	0(0-150.18)	53	
Nephritis, Nephrotic Syndrome and Nephrosis	0	0(0-8309.02)	0	0(0-9387.54)	0	0(0-4095.2)	0	0(0-1405.89)	0	0(0-845.77)	53	
Accidents and Adverse Effects	0	0(0-163.88)	0	0(0-192.44)	0	0(0-86.41)	0	0(0-54.5)	0	0(0-24.26)	53	
Suicide and Self-Inflicted Injury	0	0(0-410.54)	0	0(0-480.38)	1	58.11^{p}(1.47-323.78)	1	35.94(0.91-200.23)	2	32.42^{p}(3.93-117.09)	53	41.5
Other Cause of Death	0	0(0-261.45)	0	0(0-307.09)	0	0(0-138.63)	0	0(0-70.62)	0	0(0-35.14)	53	

Table S1: Standardized-mortality ratios (SMRs) for non-cancer causes for cardia in patients aged <39 years.

Causes	<1 years		1-2 years		2-5 years		>5 years		Total		Total	
	Observed (n)	SMR (95%CI)	Observed (n)	SMR (95%CI)	Observed (n)	Observed (n)	SMR (95%CI)	Observed (n)	SMR (95%CI)	Observed (n)	Patient	Mean Age at Event
Non-GC	104	28.30^{P}(23.13-34.29)	107	33.14^{P}(27.16-40.04)	123	15.45^{P}(12.84-18.44)	50	4.02^{P}(2.98-5.3)	384	14.07^{P}(12.7-15.55)	1521	60.01
GC	75	955.82^{P}(751.81-1198.12)	43	637.20^{P}(461.14-858.3)	43	266.04^{P}(192.54-358.36)	11	46.87^{P}(23.4-83.87)	172	317.20^{P}(271.56-368.3)	1521	58.72
All Causes of Death	219	18.17^{P}(15.85-20.75)	171	16.38^{P}(14.02-19.03)	202	7.95^{P}(6.89-9.12)	105	2.57^{P}(2.1-3.11)	697	7.85^{P}(7.28-8.45)	1521	60.28
Non-cancer causes	40	4.82^{P}(3.44-6.56)	21	2.94^{P}(1.82-4.5)	36	2.08^{P}(1.46-2.88)	44	1.56^{P}(1.13-2.09)	141	2.31^{P}(1.95-2.73)	1521	62.94
Septicemia	3	18.59^{P}(3.83-54.33)	0	0(0-25.78)	1	2.79(0.07-15.52)	0	0(0-6.16)	4	3.17(0.86-8.11)	1521	64.75
Other Infectious and Parasitic Diseases including HIV	1	4.11(0.1-22.89)	2	10.41^{P}(1.26-37.59)	2	4.9(0.59-17.69)	1	2.11(0.05-11.75)	6	4.55^{P}(1.67-9.9)	1521	63.36
Diabetes Mellitus	2	4.42(0.54-15.98)	0	0(0-9.33)	5	5.10^{P}(1.66-11.91)	4	2.52(0.69-6.46)	11	3.22^{P}(1.61-5.77)	1521	67.14
Alzheimer's (ICD-9 and 10 only)	0	0(0-128.68)	0	0(0-118.94)	0	0(0-37.74)	0	0(0-9.23)	0	0(0-6.62)	1521	
Cardiovascular Diseases	7	2.28(0.92-4.7)	3	1.13(0.23-3.32)	8	1.25(0.54-2.46)	11	1.06(0.53-1.91)	29	1.29(0.87-1.86)	1521	64.24
Cerebrovascular Diseases	1	2.63(0.07-14.65)	0	0(0-10.97)	1	1.19(0.03-6.63)	3	1.97(0.41-5.77)	5	1.63(0.53-3.79)	1521	66.7
Pneumonia and Influenza	4	26.43^{P}(7.2-67.66)	0	0(0-27.33)	1	2.92(0.07-16.24)	1	1.6(0.04-8.93)	6	4.79^{P}(1.76-10.42)	1521	59.93
Chronic Obstructive Pulmonary Disease and Allied Cond	5	9.55^{P}(3.1-22.29)	3	6.06^{P}(1.25-17.72)	2	1.5(0.18-5.43)	5	1.89(0.61-4.42)	15	3.01^{P}(1.68-4.96)	1521	64.29
Chronic Liver Disease and Cirrhosis	1	2.19(0.06-12.19)	1	2.68(0.07-14.92)	0	0(0-4.38)	1	0.97(0.02-5.38)	3	1.11(0.23-3.24)	1521	61.36
Nephritis, Nephrotic Syndrome and Nephrosis	2	12.58^{P}(1.52-45.45)	0	0(0-25.82)	1	2.73(0.07-15.22)	1	1.51(0.04-8.41)	4	3.01(0.82-7.7)	1521	53.38
Accidents and Adverse Effects	1	1.36(0.03-7.57)	4	6.75^{P}(1.84-17.28)	3	2.3(0.47-6.72)	8	4.86^{P}(2.1-9.57)	16	3.74^{P}(2.14-6.07)	1521	61.6
Suicide and Self-Inflicted Injury	4	11.82^{P}(3.22-30.27)	0	0(0-13.7)	0	0(0-6.34)	1	1.48(0.04-8.23)	5	2.68(0.87-6.25)	1521	57.54
Other Cause of Death	9	5.62^{P}(2.57-10.67)	8	5.75^{P}(2.48-11.33)	12	3.49^{P}(1.8-6.1)	8	1.32(0.57-2.61)	37	2.97^{P}(2.09-4.09)	1521	62.32

Table S2: Standardized-mortality ratios (SMRs) for non-cancer causes for cardia in patients aged 40-65 years.

Causes	<1 years		1-2 years		2-5 years		>5 years		Total		Total	
	Observed (n)	SMR (95%CI)	Observed (n)	SMR (95%CI)	Observed (n)	Observed (n)	SMR (95%CI)	Observed (n)	SMR (95%CI)	Observed (n)	Patient	Mean Age at Event
Non-GC	395	16.53[p](14.94-18.24)	181	11.06[p](9.51-12.8)	211	6.70[p](5.83-7.67)	72	2.43[p](1.9-3.07)	859	8.48[p](7.92-9.07)	2810	79.03
GC	268	546.21[p](482.76-615.67)	109	326.61[p](268.18-393.99)	83	133.79[p](106.56-165.85)	24	43.03[p](27.57-64.03)	484	241.70[p](220.64-264.22)	2810	80.59
All Causes of Death	836	7.88[p](7.36-8.44)	376	5.25[p](4.73-5.81)	453	3.17[p](2.88-3.47)	249	1.65[p](1.45-1.86)	1914	4.06[p](3.88-4.24)	2810	79.85
Non-cancer causes	173	2.12[p](1.81-2.46)	86	1.57[p](1.25-1.93)	159	1.43[p](1.22-1.67)	153	1.26[p](1.07-1.48)	571	1.55[p](1.42-1.68)	2810	80.47
Septicemia	11	7.28[p](3.64-13.03)	1	0.97(0.02-5.42)	2	0.98(0.12-3.54)	2	0.95(0.12-3.44)	16	2.39[p](1.37-3.89)	2810	74.51
Other Infectious and Parasitic Diseases including HIV	1	1.35(0.03-7.55)	2	3.97(0.48-14.33)	0	0(0-3.79)	2	2.17(0.26-7.84)	5	1.59(0.52-3.72)	2810	81.43
Diabetes Mellitus	6	1.94(0.71-4.22)	4	1.89(0.52-4.84)	1	0.24(0.01-1.35)	4	0.99(0.27-2.54)	15	1.12(0.63-1.85)	2810	80.08
Alzheimer's (ICD-9 and 10 only)	2	0.49(0.06-1.76)	2	0.73(0.09-2.63)	10	1.65(0.79-3.04)	7	0.88(0.35-1.81)	21	1.01(0.62-1.54)	2810	84.9
Cardiovascular Diseases	72	2.31[p](1.8-2.9)	19	0.92(0.55-1.43)	60	1.46[p](1.11-1.88)	58	1.32[p](1-1.71)	209	1.53[p](1.33-1.75)	2810	81.65
Cerebrovascular Diseases	9	1.49(0.68-2.83)	5	1.25(0.41-2.93)	6	0.75(0.28-1.63)	7	0.79(0.32-1.63)	27	1(0.66-1.46)	2810	82.73
Pneumonia and Influenza	1	0.37(0.01-2.08)	4	2.27(0.62-5.81)	5	1.43(0.46-3.34)	3	0.81(0.17-2.36)	13	1.12(0.59-1.91)	2810	78.94
Chronic Obstructive Pulmonary Disease and Allied Cond	14	1.98[p](1.08-3.32)	14	2.88[p](1.58-4.84)	16	1.65(0.94-2.68)	21	2.13[p](1.32-3.26)	65	2.07[p](1.59-2.63)	2810	80.5
Chronic Liver Disease and Cirrhosis	1	1.32(0.03-7.35)	3	5.65[p](1.16-16.5)	3	3.03(0.63-8.86)	1	1.22(0.03-6.79)	8	2.58[p](1.11-5.08)	2810	77.22
Nephritis, Nephrotic Syndrome and Nephrosis	2	0.89(0.11-3.21)	6	3.94[p](1.44-8.57)	9	2.95[p](1.35-5.6)	6	1.89(0.69-4.11)	23	2.30[p](1.46-3.45)	2810	79.76
Accidents and Adverse Effects	7	2.62[p](1.05-5.39)	1	0.55(0.01-3.05)	6	1.61(0.59-3.5)	3	0.72(0.15-2.11)	17	1.37(0.8-2.2)	2810	79.01
Suicide and Self-Inflicted Injury	5	8.80[p](2.86-20.54)	2	5.04(0.61-18.21)	0	0(0-4.84)	2	2.89(0.35-10.45)	9	3.72[p](1.7-7.06)	2810	76.3
Other Cause of Death	42	2.22[p](1.6-3)	23	1.78[p](1.13-2.67)	41	1.52[p](1.09-2.07)	37	1.19(0.84-1.64)	143	1.59[p](1.34-1.88)	2810	79.22

Table S3: Standardized-mortality ratios (SMRs) for non-cancer causes for cardia in patients aged >65 years.

Causes	<1 years		1-2 years		2-5 years		>5 years		Total		Total	
	Observe d (n)	SMR (95%CI)	Observe d (n)	SMR (95%CI)	Observe d (n)	Observed (n)	SMR (95%CI)	Observed (n)	SMR (95%CI)	Observed (n)	Patie nt	Mean Age at Event
Non-GC	404	17.72[p](16.04-19.54)	237	14.59[p](12.79-16.57)	280	8.56[p](7.59-9.62)	94	2.70[p](2.18-3.3)	1015	9.52[p](8.94-10.12)	3419	72.26
GC	239	493.92[p](433.28-560.67)	109	317.40[p](260.62-382.88)	97	144.43[p](117.12-176.19)	26	38.28[p](25-56.08)	471	216.23[p](197.14-236.67)	3419	72.87
All Causes of Death	806	8.75[p](8.15-9.37)	439	6.77[p](6.15-7.43)	526	3.95[p](3.62-4.3)	268	1.78[p](1.57-2)	2039	4.62[p](4.42-4.83)	3419	73.44
Non-cancer causes	163	2.37[p](2.02-2.76)	93	1.93[p](1.55-2.36)	149	1.49[p](1.26-1.75)	148	1.28[p](1.09-1.51)	553	1.66[p](1.53-1.81)	3419	76.11
Septicemia	12	9.34[p](4.82-16.31)	1	1.1(0.03-6.13)	2	1.07(0.13-3.86)	0	0(0-1.75)	15	2.43[p](1.36-4.01)	3419	71.62
Other Infectious and Parasitic Diseases including HIV	2	2.52(0.31-9.12)	3	5.30[p](1.09-15.48)	2	1.79(0.22-6.45)	3	2.67(0.55-7.81)	10	2.78[p](1.33-5.11)	3419	72.08
Diabetes Mellitus	6	2.11(0.77-4.58)	4	1.97(0.54-5.03)	4	0.96(0.26-2.46)	6	1.3(0.48-2.82)	20	1.46(0.89-2.26)	3419	74.86
Alzheimer's (ICD-9 and 10 only)	1	0.39(0.01-2.18)	2	1.13(0.14-4.08)	6	1.53(0.56-3.32)	5	0.95(0.31-2.21)	14	1.03(0.57-1.74)	3419	84.88
Cardiovascular Diseases	56	2.09[p](1.58-2.71)	20	1.08(0.66-1.66)	53	1.40[p](1.05-1.83)	54	1.26(0.95-1.64)	183	1.45[p](1.25-1.68)	3419	78.54
Cerebrovascular Diseases	8	1.76(0.76-3.47)	5	1.59(0.52-3.72)	5	0.78(0.25-1.81)	4	0.54(0.15-1.37)	22	1.02(0.64-1.54)	3419	78.55
Pneumonia and Influenza	4	1.86(0.51-4.77)	3	2.04(0.42-5.97)	4	1.34(0.36-3.43)	3	0.89(0.18-2.61)	14	1.41(0.77-2.36)	3419	69.05
Chronic Obstructive Pulmonary Disease and Allied Cond	12	2.00[p](1.03-3.5)	16	3.77[p](2.15-6.12)	16	1.83[p](1.05-2.97)	22	2.24[p](1.41-3.4)	66	2.29[p](1.77-2.92)	3419	76.99
Chronic Liver Disease and Cirrhosis	2	1.87(0.23-6.75)	4	5.02[p](1.37-12.84)	2	1.24(0.15-4.48)	2	1.22(0.15-4.42)	10	1.96(0.94-3.6)	3419	72.68
Nephritis, Nephrotic Syndrome and Nephrosis	3	1.58(0.33-4.61)	2	1.51(0.18-5.46)	8	2.94[p](1.27-5.79)	6	1.95(0.72-4.25)	19	2.11[p](1.27-3.29)	3419	76.03
Accidents and Adverse Effects	7	2.48(1-5.1)	5	2.46(0.8-5.74)	6	1.42(0.52-3.1)	6	1.25(0.46-2.73)	24	1.73[p](1.11-2.58)	3419	69.7
Suicide and Self-Inflicted Injury	9	10.34[p](4.73-19.64)	2	3.13(0.38-11.3)	1	0.77(0.02-4.32)	4	3.01(0.82-7.71)	16	3.88[p](2.22-6.29)	3419	66.09
Other Cause of Death	41	2.69[p](1.93-3.65)	26	2.41[p](1.58-3.53)	40	1.75[p](1.25-2.38)	33	1.19(0.82-1.67)	140	1.83[p](1.54-2.16)	3419	75.4

Table S4: Standardized-mortality ratios (SMRs) for non-cancer causes for cardia in male patients.

Causes	<1 years		1-2 years		2-5 years		>5 years		Total		Total	
	Observed (n)	SMR (95%CI)	Observed (n)	SMR (95%CI)	Observed (n)	Observed (n)	SMR (95%CI)	Observed (n)	SMR (95%CI)	Observed (n)	Patient	Mean Age at Event
Non-GC	99	20.68[p](16.81-25.18)	52	15.51[p](11.58-20.34)	56	8.32[p](6.28-10.8)	29	4.02[p](2.69-5.78)	236	10.69[p](9.37-12.14)	965	75.81
GC	108	1,262.71[p](1035.82-1524.52)	45	774.92[p](565.23-1036.91)	31	279.05[p](189.6-396.09)	10	87.13[p](41.78-160.23)	194	525.08[p](453.79-604.4)	965	77.99
All Causes of Death	257	9.88[p](8.71-11.17)	111	6.45[p](5.31-7.77)	134	3.80[p](3.18-4.5)	89	2.13[p](1.71-2.62)	591	4.91[p](4.52-5.32)	965	77.57
Non-cancer causes	50	2.37[p](1.76-3.12)	14	1.02(0.56-1.7)	47	1.65[p](1.21-2.2)	50	1.45[p](1.08-1.91)	161	1.64[p](1.4-1.92)	965	79.64
Septicemia	2	5.17(0.63-18.67)	0	0(0-13.99)	1	1.88(0.05-10.48)	2	3.37(0.41-12.19)	5	2.82(0.91-6.57)	965	75.37
Other Infectious and Parasitic Diseases including HIV	0	0(0-19.34)	1	7.62(0.19-42.48)	0	0(0-13.97)	0	0(0-13.23)	1	1.16(0.03-6.44)	965	66.58
Diabetes Mellitus	2	2.84(0.34-10.27)	0	0(0-7.74)	2	2.14(0.26-7.74)	2	1.99(0.24-7.18)	6	1.92(0.71-4.19)	965	73.77
Alzheimer's (ICD-9 and 10 only)	1	0.63(0.02-3.52)	0	0(0-3.67)	4	1.8(0.49-4.62)	2	0.64(0.08-2.32)	7	0.88(0.36-1.82)	965	84.95
Cardiovascular Diseases	23	3.08[p](1.95-4.62)	2	0.42(0.05-1.52)	15	1.56(0.87-2.57)	15	1.32(0.74-2.18)	55	1.65[p](1.25-2.15)	965	82.83
Cerebrovascular Diseases	2	1.07(0.13-3.87)	0	0(0-3.1)	2	0.83(0.1-3)	6	2.05(0.75-4.46)	10	1.19(0.57-2.19)	965	83.93
Pneumonia and Influenza	1	1.46(0.04-8.14)	1	2.32(0.06-12.93)	2	2.35(0.28-8.5)	1	1.01(0.03-5.65)	5	1.69(0.55-3.95)	965	83.83
Chronic Obstructive Pulmonary Disease and Allied Cond	7	4.37[p](1.76-9.01)	1	0.91(0.02-5.05)	2	0.88(0.11-3.18)	4	1.49(0.41-3.82)	14	1.83(1-3.07)	965	79.68
Chronic Liver Disease and Cirrhosis	0	0(0-25.09)	0	0(0-33.82)	1	4.42(0.11-24.65)	0	0(0-15.46)	1	1.39(0.04-7.73)	965	75
Nephritis, Nephrotic Syndrome and Nephrosis	1	1.97(0.05-10.96)	4	11.64[p](3.17-29.81)	2	2.89(0.35-10.45)	1	1.3(0.03-7.23)	8	3.46[p](1.49-6.81)	965	75.43
Accidents and Adverse Effects	1	1.65(0.04-9.17)	0	0(0-9.1)	3	3.46(0.71-10.1)	5	4.62[p](1.5-10.78)	9	3.04[p](1.39-5.77)	965	72.9
Suicide and Self-Inflicted Injury	0	0(0-81.27)	0	0(0-108.29)	0	0(0-52.07)	0	0(0-54.39)	0	0(0-16.91)	965	
Other Cause of Death	10	1.88(0.9-3.45)	5	1.42(0.46-3.31)	13	1.73(0.92-2.96)	12	1.28(0.66-2.23)	40	1.55[p](1.11-2.11)	965	76.94

Table S5: Standardized-mortality ratios (SMRs) for non-cancer causes for cardia in female patients.

Causes	<1 years		1-2 years		2-5 years		>5 years		Total		Total	
	Observed (n)	SMR (95%CI)	Observed (n)	SMR (95%CI)	Observed (n)	Observed (n)	SMR (95%CI)	Observed (n)	SMR (95%CI)	Observed (n)	Patient	Mean Age at Event
Non-GC	457	18.27^{p}(16.63-20.02)	266	14.96^{p}(13.22-16.87)	318	8.84^{p}(7.9-9.87)	109	2.79^{p}(2.29-3.37)	1150	9.76^{p}(9.21-10.34)	3886	72.98
GC	288	640.30^{p}(568.48-718.68)	121	383.18^{p}(317.95-457.85)	99	158.32^{p}(128.67-192.75)	28	42.68^{p}(28.36-61.69)	536	261.86^{p}(240.16-285)	3886	74.25
All Causes of Death	934	8.78^{p}(8.23-9.36)	482	6.56^{p}(5.98-7.17)	590	3.88^{p}(3.57-4.21)	318	1.78^{p}(1.59-1.99)	2324	4.55^{p}(4.37-4.74)	3886	74.31
Non-cancer causes	189	2.34^{p}(2.02-2.69)	95	1.71^{p}(1.39-2.1)	173	1.50^{p}(1.28-1.74)	181	1.31^{p}(1.12-1.51)	638	1.63^{p}(1.51-1.77)	3886	76.77
Septicemia	11	7.51^{p}(3.75-13.43)	1	0.98(0.02-5.44)	2	0.94(0.11-3.4)	2	0.81(0.1-2.92)	16	2.26^{p}(1.29-3.66)	3886	71.58
Other Infectious and Parasitic Diseases including HIV	2	2.36(0.29-8.53)	3	4.99^{p}(1.03-14.58)	1	0.83(0.02-4.62)	3	2.39(0.49-6.98)	9	2.30^{p}(1.05-4.37)	3886	73
Diabetes Mellitus	6	1.98(0.73-4.3)	4	1.87(0.51-4.78)	4	0.91(0.25-2.34)	7	1.4(0.56-2.89)	21	1.44(0.89-2.21)	3886	73.79
Alzheimer's (ICD-9 and 10 only)	1	0.27(0.01-1.48)	2	0.8(0.1-2.9)	9	1.62(0.74-3.07)	7	0.89(0.36-1.84)	19	0.97(0.58-1.51)	3886	84.65
Cardiovascular Diseases	74	2.40^{p}(1.89-3.01)	21	1.01(0.62-1.54)	59	1.38^{p}(1.05-1.78)	63	1.25(0.96-1.6)	217	1.50^{p}(1.31-1.71)	3886	79.36
Cerebrovascular Diseases	7	1.25(0.5-2.58)	3	0.8(0.17-2.35)	5	0.65(0.21-1.51)	7	0.75(0.3-1.54)	22	0.83(0.52-1.26)	3886	79.42
Pneumonia and Influenza	4	1.62(0.44-4.16)	4	2.46(0.67-6.29)	6	1.81(0.66-3.94)	4	1.02(0.28-2.62)	18	1.59(0.94-2.52)	3886	72.76
Chronic Obstructive Pulmonary Disease and Allied Cond	19	2.68^{p}(1.61-4.18)	14	2.81^{p}(1.53-4.71)	17	1.65(0.96-2.64)	23	1.94^{p}(1.23-2.9)	73	2.13^{p}(1.67-2.68)	3886	77.27
Chronic Liver Disease and Cirrhosis	2	1.77(0.21-6.4)	4	4.75^{p}(1.29-12.16)	3	1.75(0.36-5.12)	2	1.13(0.14-4.1)	11	2.02^{p}(1.01-3.61)	3886	72.89
Nephritis, Nephrotic Syndrome and Nephrosis	3	1.44(0.3-4.22)	5	3.50^{p}(1.14-8.17)	9	3.04^{p}(1.39-5.78)	6	1.73(0.64-3.77)	23	2.32^{p}(1.47-3.48)	3886	76.12
Accidents and Adverse Effects	8	2.55^{p}(1.1-5.02)	4	1.8(0.49-4.61)	9	1.94(0.89-3.67)	10	1.83(0.88-3.36)	31	2.00^{p}(1.36-2.84)	3886	71
Suicide and Self-Inflicted Injury	8	9.12^{p}(3.94-17.98)	2	3.1(0.38-11.19)	1	0.77(0.02-4.27)	4	2.97(0.81-7.61)	15	3.59^{p}(2.01-5.93)	3886	66.81
Other Cause of Death	44	2.37^{p}(1.72-3.18)	28	2.18^{p}(1.45-3.15)	48	1.74^{p}(1.29-2.31)	43	1.24(0.9-1.67)	163	1.74^{p}(1.49-2.03)	3886	75.76

Table S6: Standardized-mortality ratios (SMRs) for non-cancer causes for cardia in white patients.

Causes	<1 years		1-2 years		2-5 years		>5 years		Total		Total	
	Observed (n)	SMR (95%CI)	Observed (n)	SMR (95%CI)	Observed (n)	Observed (n)	SMR (95%CI)	Observed (n)	SMR (95%CI)	Observed (n)	Patient	Mean Age at Event
Non-GC	22	17.02[P](10.67-25.77)	12	13.80[P](7.13-24.1)	7	4.36[P](1.75-8.99)	7	6.13[P](2.46-12.62)	48	9.78[P](7.21-12.96)	203	70.12
GC	35	790.94[P](550.92-1100)	11	374.84[P](187.12-670.7)	11	209.93[P](104.79-375.62)	2	53.87[P](6.52-194.59)	59	361.69[P](275.34-466.55)	203	73.27
All Causes of Death	71	12.16[P](9.5-15.34)	30	7.30[P](4.93-10.42)	28	3.63[P](2.41-5.25)	15	2.69[P](1.51-4.44)	144	6.20[P](5.23-7.3)	203	72.51
Non-cancer causes	14	3.11[P](1.7-5.22)	7	2.18(0.88-4.49)	10	1.65(0.79-3.04)	6	1.37(0.5-2.97)	37	2.04[P](1.44-2.81)	203	74.42
Septicemia	2	15.40[P](1.86-55.62)	0	0(0-40.93)	1	6.05(0.15-33.69)	0	0(0-32.02)	3	5.99[P](1.24-17.51)	203	72.08
Other Infectious and Parasitic Diseases including HIV	0	0(0-51.51)	0	0(0-76.48)	0	0(0-44.28)	0	0(0-70.22)	0	0(0-14.43)	203	
Diabetes Mellitus	0	0(0-14.13)	0	0(0-20.54)	2	5.99(0.73-21.65)	0	0(0-15.16)	2	1.97(0.24-7.1)	203	67.22
Alzheimer's (ICD-9 and 10 only)	1	5.46(0.14-30.4)	0	0(0-24.82)	1	3.27(0.08-18.23)	0	0(0-15.32)	2	2.28(0.28-8.23)	203	87.29
Cardiovascular Diseases	3	1.7(0.35-4.98)	1	0.81(0.02-4.49)	5	2.17(0.7-5.06)	3	1.82(0.38-5.33)	12	1.73(0.89-3.01)	203	79.31
Cerebrovascular Diseases	2	5.32(0.64-19.21)	1	3.74(0.09-20.82)	0	0(0-7.31)	3	7.92[P](1.63-23.13)	6	3.93[P](1.44-8.55)	203	79.75
Pneumonia and Influenza	0	0(0-29.03)	0	0(0-41.62)	0	0(0-22.28)	0	0(0-32.36)	0	0(0-7.45)	203	
Chronic Obstructive Pulmonary Disease and Allied Cond	0	0(0-16.35)	1	6.47(0.16-36.05)	1	3.36(0.09-18.71)	0	0(0-16.64)	2	2.22(0.27-8.03)	203	68.04
Chronic Liver Disease and Cirrhosis	0	0(0-102.22)	0	0(0-144.83)	0	0(0-79.06)	0	0(0-119.42)	0	0(0-26.52)	203	
Nephritis, Nephrotic Syndrome and Nephrosis	1	5.21(0.13-29)	1	7.44(0.19-41.45)	0	0(0-14.66)	0	0(0-20.24)	2	2.63(0.32-9.5)	203	64.83
Accidents and Adverse Effects	0	0(0-30.48)	1	11.66(0.3-64.98)	0	0(0-22.11)	0	0(0-30.63)	1	2.02(0.05-11.28)	203	55
Suicide and Self-Inflicted Injury	1	97.96[P](2.48-545.8)	0	0(0-550.5)	0	0(0-307.97)	0	0(0-461.95)	1	27.12(0.69-151.1)	203	55.25
Other Cause of Death	4	3.97[P](1.08-10.17)	2	2.71(0.33-9.78)	0	0(0-2.61)	0	0(0-3.56)	6	1.43(0.52-3.11)	203	70.34

Table S7: Standardized-mortality ratios (SMRs) for non-cancer causes for cardia in black patients.

Causes	<1 years		1-2 years		2-5 years		>5 years		Total		Total	
	Observed (n)	SMR (95%CI)	Observed (n)	SMR (95%CI)	Observed (n)	Observed (n)	SMR (95%CI)	Observed (n)	SMR (95%CI)	Observed (n)	Patient	Mean Age at Event
Non-GC	2	32.57[p](3.94-117.65)	0	0(0-73.64)	1	10.48(0.27-58.38)	0	0(0-39.39)	3	9.98[p](2.06-29.17)	21	67.39
GC	2	527.28[p](63.86-1904.73)	0	0(0-1314.15)	1	206.01[p](5.22-1147.83)	1	212.25[p](5.37-1182.61)	4	247.44[p](67.42-633.55)	21	79.33
All Causes of Death	5	22.90[p](7.43-53.43)	2	11.51[p](1.39-41.56)	5	15.56[p](5.05-36.32)	1	2.93(0.07-16.32)	13	12.32[p](6.56-21.07)	21	71.86
Non-cancer causes	1	6.53(0.17-36.37)	2	16.54[p](2-59.74)	3	13.58[p](2.8-39.68)	0	0(0-15.18)	6	8.13[p](2.98-17.69)	21	69.11
Septicemia	0	0(0-1280.1)	0	0(0-1484.48)	0	0(0-800.61)	0	0(0-806.32)	0	0(0-253.55)	21	
Other Infectious and Parasitic Diseases including HIV	0	0(0-1212.34)	1	397.98[p](10.08-2217.42)	1	240.25[p](6.08-1338.59)	0	0(0-921.43)	2	145.76[p](17.65-526.54)	21	65.17
Diabetes Mellitus	0	0(0-345.92)	0	0(0-419.89)	0	0(0-216.66)	0	0(0-205.21)	0	0(0-67.75)	21	
Alzheimer's (ICD-9 and 10 only)	0	0(0-1752.43)	0	0(0-2191.38)	0	0(0-1177.84)	0	0(0-662.58)	0	0(0-295.4)	21	
Cardiovascular Diseases	1	16.94(0.43-94.38)	0	0(0-79.89)	0	0(0-44.69)	0	0(0-42.34)	1	3.64(0.09-20.27)	21	70.33
Cerebrovascular Diseases	0	0(0-255.21)	0	0(0-337.45)	0	0(0-185.25)	0	0(0-161.73)	0	0(0-54.16)	21	
Pneumonia and Influenza	0	0(0-580.17)	0	0(0-723.4)	0	0(0-410.05)	0	0(0-393.78)	0	0(0-123.7)	21	
Chronic Obstructive Pulmonary Disease and Allied Cond	0	0(0-384.44)	1	140.50[p](3.56-782.8)	0	0(0-297.51)	0	0(0-243.78)	1	22.6(0.57-125.93)	21	72.08
Chronic Liver Disease and Cirrhosis	0	0(0-1032.93)	0	0(0-1262.54)	0	0(0-728.55)	0	0(0-1007.91)	0	0(0-242.43)	21	
Nephritis, Nephrotic Syndrome and Nephrosis	0	0(0-812.81)	0	0(0-998.71)	0	0(0-521.25)	0	0(0-446.88)	0	0(0-156.55)	21	
Accidents and Adverse Effects	0	0(0-487.4)	0	0(0-679.41)	0	0(0-362.64)	0	0(0-346.63)	0	0(0-109.1)	21	
Suicide and Self-Inflicted Injury	0	0(0-2006.3)	0	0(0-2721.55)	0	0(0-1410.15)	0	0(0-1819.09)	0	0(0-470.65)	21	
Other Cause of Death	0	0(0-133.99)	0	0(0-162.17)	2	46.23[p](5.6-167)	0	0(0-71.08)	2	13.75[p](1.67-49.68)	21	70.96

Table S8: Standardized-mortality ratios (SMRs) for non-cancer causes for cardia in American Indian/Alaska Native patients.

Causes	<1 years		1-2 years		2-5 years		>5 years		Total		Total	
	Observe d (n)	SMR (95%CI)	Observe d (n)	SMR (95%CI)	Observe d (n)	Observed (n)	SMR (95%CI)	Observed (n)	SMR (95%CI)	Observed (n)	Pati ent	Mean Age at Event
Non-GC	22	18.16^{p}(11.38-27.5)	11	12.21^{p}(6.1-21.85)	10	5.60^{p}(2.68-10.29)	7	3.89^{p}(1.57-8.02)	50	8.78^{p}(6.52-11.57)	274	74.88
GC	22	307.35^{p}(192.61-465.33)	22	410.78^{p}(257.43-621.92)	17	169.76^{p}(98.89-271.81)	5	51.96^{p}(16.87-121.26)	66	205.29^{p}(158.77-261.17)	274	75.93
All Causes of Death	53	9.19^{p}(6.88-12.02)	36	8.40^{p}(5.89-11.63)	37	4.38^{p}(3.08-6.03)	23	2.76^{p}(1.75-4.15)	149	5.55^{p}(4.7-6.52)	274	77.28
Non-cancer causes	9	2.01(0.92-3.81)	3	0.9(0.19-2.63)	10	1.52(0.73-2.8)	11	1.71(0.85-3.06)	33	1.59^{p}(1.09-2.23)	274	83.62
Septicemia	1	13.5(0.34-75.23)	0	0(0-67.08)	0	0(0-34.03)	0	0(0-35.12)	1	2.92(0.07-16.27)	274	89.67
Other Infectious and Parasitic Diseases including HIV	0	0(0-60.13)	0	0(0-81.21)	0	0(0-40.77)	0	0(0-42.12)	0	0(0-12.95)	274	
Diabetes Mellitus	2	8.19(0.99-29.59)	0	0(0-20.39)	0	0(0-10.18)	1	2.67(0.07-14.9)	3	2.58(0.53-7.55)	274	85.28
Alzheimer's (ICD-9 and 10 only)	0	0(0-19.55)	0	0(0-27.26)	0	0(0-13.32)	0	0(0-12.06)	0	0(0-4.07)	274	
Cardiovascular Diseases	1	0.61(0.02-3.37)	0	0(0-3.02)	4	1.68(0.46-4.29)	3	1.32(0.27-3.85)	8	1.06(0.46-2.09)	274	85.76
Cerebrovascular Diseases	1	2.28(0.06-12.72)	1	3.14(0.08-17.49)	2	3.29(0.4-11.88)	0	0(0-6.06)	4	2.03(0.55-5.19)	274	85.41
Pneumonia and Influenza	1	4.27(0.11-23.78)	0	0(0-20.84)	0	0(0-10.54)	0	0(0-11.83)	1	0.93(0.02-5.19)	274	76.08
Chronic Obstructive Pulmonary Disease and Allied Cond	0	0(0-13.97)	1	4.96(0.13-27.64)	0	0(0-9.29)	3	8.08^{p}(1.67-23.6)	4	3.24(0.88-8.3)	274	87.04
Chronic Liver Disease and Cirrhosis	0	0(0-76.49)	0	0(0-102.14)	0	0(0-50.88)	0	0(0-49.66)	0	0(0-15.96)	274	
Nephritis, Nephrotic Syndrome and Nephrosis	0	0(0-27.63)	0	0(0-36.72)	1	4.98(0.13-27.76)	1	5.13(0.13-28.56)	2	3.18(0.38-11.47)	274	83.79
Accidents and Adverse Effects	0	0(0-22.19)	0	0(0-29.55)	0	0(0-14.49)	1	3.93(0.1-21.91)	1	1.25(0.03-6.96)	274	72.83
Suicide and Self-Inflicted Injury	0	0(0-138.77)	0	0(0-186.49)	0	0(0-89.7)	0	0(0-93.72)	0	0(0-29.08)	274	
Other Cause of Death	3	3.14(0.65-9.19)	1	1.4(0.04-7.82)	3	2.11(0.44-6.17)	2	1.4(0.17-5.06)	9	1.99(0.91-3.78)	274	80.17

Table S9: Standardized-mortality ratios (SMRs) for non-cancer causes for cardia in Asian/Pacific Islander patients.

Causes	<1 years		1-2 years		2-5 years		>5 years		Total		Total	
	Observed (n)	SMR (95%CI)	Observed (n)	SMR (95%CI)	Observed (n)	Observed (n)	SMR (95%CI)	Observed (n)	SMR (95%CI)	Observed (n)	Patie nt	Mean Age at Event
Non-GC	0	0(0-0)	0	0(0-0)	0	0(0-0)	0	0(0-0)	0	0(0-0)	0	0
GC	0	0(0-0)	0	0(0-0)	0	0(0-0)	0	0(0-0)	0	0(0-0)	0	0
All Causes of Death	0	0(0-0)	0	0(0-0)	0	0(0-0)	0	0(0-0)	0	0(0-0)	0	0
Non-cancer causes	0	0(0-0)	0	0(0-0)	0	0(0-0)	0	0(0-0)	0	0(0-0)	0	0
Septicemia	0	0(0-0)	0	0(0-0)	0	0(0-0)	0	0(0-0)	0	0(0-0)	0	0
Other Infectious and Parasitic Diseases including HIV	0	0(0-0)	0	0(0-0)	0	0(0-0)	0	0(0-0)	0	0(0-0)	0	0
Diabetes Mellitus	0	0(0-0)	0	0(0-0)	0	0(0-0)	0	0(0-0)	0	0(0-0)	0	0
Alzheimer's (ICD-9 and 10 only)	0	0(0-0)	0	0(0-0)	0	0(0-0)	0	0(0-0)	0	0(0-0)	0	0
Cardiovascular Diseases	0	0(0-0)	0	0(0-0)	0	0(0-0)	0	0(0-0)	0	0(0-0)	0	0
Cerebrovascular Diseases	0	0(0-0)	0	0(0-0)	0	0(0-0)	0	0(0-0)	0	0(0-0)	0	0
Pneumonia and Influenza	0	0(0-0)	0	0(0-0)	0	0(0-0)	0	0(0-0)	0	0(0-0)	0	0
Chronic Obstructive Pulmonary Disease and Allied Cond	0	0(0-0)	0	0(0-0)	0	0(0-0)	0	0(0-0)	0	0(0-0)	0	0
Chronic Liver Disease and Cirrhosis	0	0(0-0)	0	0(0-0)	0	0(0-0)	0	0(0-0)	0	0(0-0)	0	0
Nephritis, Nephrotic Syndrome and Nephrosis	0	0(0-0)	0	0(0-0)	0	0(0-0)	0	0(0-0)	0	0(0-0)	0	0
Accidents and Adverse Effects	0	0(0-0)	0	0(0-0)	0	0(0-0)	0	0(0-0)	0	0(0-0)	0	0
Suicide and Self-Inflicted Injury	0	0(0-0)	0	0(0-0)	0	0(0-0)	0	0(0-0)	0	0(0-0)	0	0
Other Cause of Death	0	0(0-0)	0	0(0-0)	0	0(0-0)	0	0(0-0)	0	0(0-0)	0	0

Table S10: Standardized-mortality ratios (SMRs) for non-cancer causes for cardia in unknown race patients.

Causes	<1 years		1-2 years		2-5 years		>5 years		Total		Total	
	Observed (n)	SMR (95%CI)	Observed (n)	SMR (95%CI)	Observed (n)	Observed (n)	SMR (95%CI)	Observed (n)	SMR (95%CI)	Observed (n)	Patient	Mean Age at Event
Non-GC	36	20.43^{P}(14.31-28.29)	20	16.83^{P}(10.28-25.99)	32	13.18^{P}(9.02-18.61)	11	2.05^{P}(1.02-3.66)	99	9.20^{P}(7.48-11.21)	258	71.65
GC	26	654.28^{P}(427.4-958.67)	14	512.03^{P}(279.93-859.1)	9	164.15^{P}(75.06-311.6)	4	37.29^{P}(10.16-95.47)	53	231.26^{P}(173.23-302.49)	258	75.14
All Causes of Death	72	9.11^{P}(7.13-11.48)	43	8.46^{P}(6.12-11.39)	55	5.44^{P}(4.1-7.08)	47	1.94^{P}(1.42-2.58)	217	4.58^{P}(3.99-5.24)	258	74.25
Non-cancer causes	10	1.64(0.79-3.01)	9	2.33^{P}(1.06-4.42)	14	1.83^{P}(1-3.08)	32	1.71^{P}(1.17-2.41)	65	1.79^{P}(1.38-2.28)	258	77.5
Septicemia	0	0(0-34.71)	0	0(0-54.5)	1	7.28(0.18-40.57)	1	2.98(0.08-16.6)	2	3.09(0.37-11.17)	258	75.54
Other Infectious and Parasitic Diseases including HIV	0	0(0-63.71)	0	0(0-87.43)	0	0(0-41.85)	1	5.41(0.14-30.14)	1	2.68(0.07-14.93)	258	61.58
Diabetes Mellitus	0	0(0-15.76)	0	0(0-23.93)	0	0(0-12.36)	2	2.89(0.35-10.45)	2	1.45(0.18-5.24)	258	79.01
Alzheimer's (ICD-9 and 10 only)	0	0(0-14.07)	0	0(0-22.67)	0	0(0-11.1)	2	1.98(0.24-7.14)	2	1.13(0.14-4.08)	258	82.75
Cardiovascular Diseases	3	1.15(0.24-3.37)	2	1.24(0.15-4.49)	5	1.65(0.53-3.84)	9	1.31(0.6-2.48)	19	1.34(0.81-2.1)	258	79.09
Cerebrovascular Diseases	2	3.83(0.46-13.82)	2	6.45(0.78-23.3)	1	1.74(0.04-9.68)	3	2.27(0.47-6.65)	8	2.93^{P}(1.27-5.78)	258	81.69
Pneumonia and Influenza	1	4.21(0.11-23.47)	0	0(0-25.67)	1	3.87(0.1-21.56)	2	3.54(0.43-12.79)	4	3.32(0.9-8.5)	258	77.43
Chronic Obstructive Pulmonary Disease and Allied Cond	1	2.1(0.05-11.68)	2	6.44(0.78-23.27)	0	0(0-5.7)	4	2.51(0.68-6.42)	7	2.31(0.93-4.76)	258	78.61
Chronic Liver Disease and Cirrhosis	0	0(0-58.08)	0	0(0-78.16)	0	0(0-37.27)	0	0(0-16.86)	0	0(0-8.61)	258	
Nephritis, Nephrotic Syndrome and Nephrosis	1	6.68(0.17-37.2)	0	0(0-38.43)	0	0(0-18.09)	1	2.06(0.05-11.5)	2	2.14(0.26-7.73)	258	77.13
Accidents and Adverse Effects	0	0(0-18.98)	1	7.56(0.19-42.12)	2	7.23(0.88-26.1)	1	1.42(0.04-7.89)	4	3.05(0.83-7.82)	258	65.77
Suicide and Self-Inflicted Injury	0	0(0-77.38)	0	0(0-105.62)	0	0(0-49.83)	1	5.96(0.15-33.23)	1	3.08(0.08-17.18)	258	81.61
Other Cause of Death	2	1.75(0.21-6.33)	2	2.64(0.32-9.54)	4	2.49(0.68-6.38)	5	1.09(0.35-2.54)	13	1.61(0.85-2.75)	258	75.83

Table S11: Standardized-mortality ratios (SMRs) for non-cancer causes for cardia during 2000-2004.

Causes	<1 years		1-2 years		2-5 years		>5 years		Total		Total	
	Observed (n)	SMR (95%CI)	Observed (n)	SMR (95%CI)	Observed (n)	Observed (n)	SMR (95%CI)	Observed (n)	SMR (95%CI)	Observed (n)	Patient	Mean Age at Event
Non-GC	159	17.85[p](15.18-20.85)	91	13.89[p](11.18-17.06)	134	9.20[p](7.71-10.89)	67	2.57[p](1.99-3.27)	451	8.04[p](7.32-8.82)	1315	72.76
GC	120	622.35[p](515.99-744.18)	47	340.40[p](250.12-452.66)	49	166.04[p](122.84-219.51)	18	36.67[p](21.73-57.95)	234	209.51[p](183.52-238.14)	1315	74.39
All Causes of Death	362	9.89[p](8.9-10.96)	170	6.37[p](5.45-7.4)	236	3.91[p](3.43-4.44)	197	1.65[p](1.43-1.9)	965	3.97[p](3.72-4.23)	1315	74.62
Non-cancer causes	83	3.02[p](2.4-3.74)	32	1.60[p](1.09-2.26)	53	1.16(0.87-1.52)	112	1.21(0.99-1.45)	280	1.51[p](1.34-1.69)	1315	77.78
Septicemia	9	17.55[p](8.03-33.32)	0	0(0-9.86)	0	0(0-4.4)	1	0.6(0.02-3.32)	10	2.94[p](1.41-5.4)	1315	73.49
Other Infectious and Parasitic Diseases including HIV	1	3.2(0.08-17.81)	1	4.28(0.11-23.86)	1	1.9(0.05-10.56)	1	1.14(0.03-6.36)	4	2.05(0.56-5.25)	1315	75.77
Diabetes Mellitus	3	2.77(0.57-8.08)	2	2.54(0.31-9.19)	0	0(0-2.09)	4	1.16(0.32-2.96)	9	1.27(0.58-2.41)	1315	75.75
Alzheimer's (ICD-9 and 10 only)	1	0.88(0.02-4.89)	0	0(0-4.38)	2	1(0.12-3.6)	2	0.39(0.05-1.4)	5	0.55(0.18-1.27)	1315	82.27
Cardiovascular Diseases	30	2.72[p](1.84-3.89)	8	1.02(0.44-2.01)	18	1.04(0.62-1.65)	38	1.12(0.8-1.54)	94	1.35[p](1.09-1.65)	1315	80.58
Cerebrovascular Diseases	2	0.98(0.12-3.53)	1	0.69(0.02-3.86)	1	0.32(0.01-1.76)	7	1.09(0.44-2.24)	11	0.84(0.42-1.5)	1315	78.36
Pneumonia and Influenza	1	1.08(0.03-6.04)	1	1.56(0.04-8.68)	2	1.41(0.17-5.11)	0	0(0-1.35)	4	0.7(0.19-1.79)	1315	69.9
Chronic Obstructive Pulmonary Disease and Allied Cond	8	3.35[p](1.45-6.61)	5	2.84(0.92-6.62)	5	1.24(0.4-2.9)	15	1.93[p](1.08-3.18)	33	2.07[p](1.43-2.91)	1315	79.58
Chronic Liver Disease and Cirrhosis	2	5.94(0.72-21.46)	1	3.84(0.1-21.42)	1	1.65(0.04-9.2)	2	1.78(0.22-6.42)	6	2.58(0.95-5.61)	1315	69.37
Nephritis, Nephrotic Syndrome and Nephrosis	0	0(0-4.75)	2	3.51(0.43-12.7)	1	0.81(0.02-4.49)	5	2.09(0.68-4.87)	8	1.61(0.69-3.16)	1315	76.05
Accidents and Adverse Effects	1	1.03(0.03-5.71)	1	1.37(0.03-7.61)	3	1.77(0.37-5.17)	5	1.4(0.45-3.27)	10	1.43(0.69-2.64)	1315	71.47
Suicide and Self-Inflicted Injury	6	22.93[p](8.42-49.92)	0	0(0-18.24)	0	0(0-7.93)	2	2.37(0.29-8.56)	8	4.51[p](1.95-8.89)	1315	65.33
Other Cause of Death	19	3.32[p](2-5.18)	10	2.31[p](1.11-4.25)	19	1.81[p](1.09-2.82)	30	1.3(0.88-1.86)	78	1.79[p](1.41-2.23)	1315	77.49

Table S12: Standardized-mortality ratios (SMRs) for non-cancer causes for cardia during 2005-2009.

Causes	<1 years		1-2 years		2-5 years		>5 years		Total		Total	
	Observed (n)	SMR (95%CI)	Observed (n)	SMR (95%CI)	Observed (n)	Observed (n)	SMR (95%CI)	Observed (n)	SMR (95%CI)	Observed (n)	Patient	Mean Age at Event
Non-GC	196	18.47[p](15.97-21.24)	127	15.87[p](13.23-18.88)	140	7.69[p](6.47-9.07)	45	4.23[p](3.09-5.66)	508	10.70[p](9.79-11.68)	1663	72.88
GC	145	663.51[p](559.91-780.72)	72	438.88[p](343.4-552.7)	57	160.37[p](121.46-207.78)	14	71.47[p](39.07-119.92)	288	308.38[p](273.79-346.14)	1663	73.98
All Causes of Death	418	9.14[p](8.28-10.06)	241	7.14[p](6.27-8.1)	289	3.62[p](3.22-4.07)	113	2.31[p](1.9-2.78)	1061	5.10[p](4.79-5.41)	1663	74.08
Non-cancer causes	77	2.20[p](1.74-2.76)	42	1.64[p](1.18-2.22)	92	1.50[p](1.21-1.84)	54	1.42[p](1.06-1.85)	265	1.66[p](1.46-1.87)	1663	76.5
Septicemia	3	4.56(0.94-13.32)	0	0(0-7.44)	2	1.71(0.21-6.16)	0	0(0-5.38)	5	1.66(0.54-3.87)	1663	71.22
Other Infectious and Parasitic Diseases including HIV	1	2.51(0.06-13.99)	1	3.4(0.09-18.93)	0	0(0-5.84)	1	2.94(0.07-16.39)	3	1.8(0.37-5.27)	1663	75.72
Diabetes Mellitus	5	3.64[p](1.18-8.49)	2	1.92(0.23-6.94)	5	2.05(0.67-4.79)	2	1.35(0.16-4.87)	14	2.21[p](1.21-3.71)	1663	73.98
Alzheimer's (ICD-9 and 10 only)	0	0(0-2.32)	2	1.74(0.21-6.29)	6	1.91(0.7-4.17)	3	1.37(0.28-4)	11	1.36(0.68-2.44)	1663	87.86
Cardiovascular Diseases	28	2.17[p](1.44-3.13)	7	0.75(0.3-1.54)	34	1.53[p](1.06-2.14)	22	1.62[p](1.01-2.45)	91	1.56[p](1.26-1.92)	1663	79.7
Cerebrovascular Diseases	3	1.26(0.26-3.67)	2	1.15(0.14-4.16)	2	0.48(0.06-1.73)	0	0(0-1.4)	7	0.64(0.26-1.32)	1663	81.4
Pneumonia and Influenza	3	2.77(0.57-8.08)	2	2.56(0.31-9.26)	0	0(0-2.05)	2	1.93(0.23-6.99)	7	1.49(0.6-3.07)	1663	68.66
Chronic Obstructive Pulmonary Disease and Allied Cond	5	1.7(0.55-3.96)	8	3.63[p](1.57-7.14)	10	1.93(0.93-3.55)	7	2.24(0.9-4.62)	30	2.23[p](1.5-3.18)	1663	76.17
Chronic Liver Disease and Cirrhosis	0	0(0-7.75)	3	7.89[p](1.63-23.06)	2	2.27(0.27-8.19)	0	0(0-6.98)	5	2.21(0.72-5.15)	1663	77.12
Nephritis, Nephrotic Syndrome and Nephrosis	1	1.06(0.03-5.93)	3	4.39(0.9-12.82)	7	4.32[p](1.74-8.91)	1	1.04(0.03-5.77)	12	2.85[p](1.47-4.98)	1663	73.52
Accidents and Adverse Effects	6	4.51[p](1.65-9.81)	1	1(0.03-5.55)	3	1.22(0.25-3.56)	5	3.15[p](1.02-7.35)	15	2.35[p](1.31-3.87)	1663	71.8
Suicide and Self-Inflicted Injury	1	2.79(0.07-15.53)	1	3.58(0.09-19.96)	0	0(0-5.72)	1	2.6(0.07-14.48)	3	1.8(0.37-5.26)	1663	62.2
Other Cause of Death	21	2.49[p](1.54-3.8)	10	1.62(0.78-2.98)	21	1.42(0.88-2.16)	10	1.05(0.5-1.93)	62	1.59[p](1.22-2.04)	1663	73.65

Table S13: Standardized-mortality ratios (SMRs) for non-cancer causes for cardia during 2010-2014.

Causes	<1 years		1-2 years		2-5 years		>5 years		Total		Total	
	Observed (n)	SMR (95%CI)	Observed (n)	SMR (95%CI)	Observed (n)	Observed (n)	SMR (95%CI)	Observed (n)	SMR (95%CI)	Observed (n)	Patient	Mean Age at Event
Non-GC	112	17.78^{p}(14.64-21.39)	51	13.23^{p}(9.85-17.39)	30	7.08^{p}(4.78-10.11)	0	0(0-0)	193	13.41^{p}(11.59-15.44)	1148	74.11
GC	56	473.27^{p}(357.5-614.58)	21	291.58^{p}(180.5-445.72)	13	168.07^{p}(89.49-287.41)	0	0(0-0)	90	336.21^{p}(270.35-413.25)	1148	75.03
All Causes of Death	211	7.56^{p}(6.57-8.65)	96	5.81^{p}(4.7-7.09)	80	4.37^{p}(3.46-5.44)	0	0(0-0)	387	6.17^{p}(5.57-6.81)	1148	74.62
Non-cancer causes	43	2.00^{p}(1.45-2.69)	24	1.90^{p}(1.22-2.83)	37	2.64^{p}(1.86-3.64)	0	0(0-0)	104	2.16^{p}(1.77-2.62)	1148	75.2
Septicemia	2	5.06(0.61-18.28)	1	4.26(0.11-23.75)	0	0(0-14.49)	0	0(0-0)	3	3.39(0.7-9.91)	1148	69.7
Other Infectious and Parasitic Diseases including HIV	0	0(0-17.24)	2	15.70^{p}(1.9-56.72)	1	7.28(0.18-40.57)	0	0(0-0)	3	6.27^{p}(1.29-18.31)	1148	65.17
Diabetes Mellitus	0	0(0-4.3)	0	0(0-6.97)	1	1.68(0.04-9.37)	0	0(0-0)	1	0.5(0.01-2.81)	1148	64.33
Alzheimer's (ICD-9 and 10 only)	1	0.87(0.02-4.84)	0	0(0-5.91)	2	2.94(0.36-10.62)	0	0(0-0)	3	1.22(0.25-3.57)	1148	79.89
Cardiovascular Diseases	18	2.32^{p}(1.38-3.67)	5	1.1(0.36-2.58)	11	2.19^{p}(1.09-3.92)	0	0(0-0)	34	1.97^{p}(1.36-2.75)	1148	76.41
Cerebrovascular Diseases	3	2.06(0.42-6.01)	0	0(0-4.4)	3	3.25(0.67-9.49)	0	0(0-0)	6	1.86(0.68-4.06)	1148	80.34
Pneumonia and Influenza	0	0(0-6.29)	1	3.01(0.08-16.76)	3	8.24^{p}(1.7-24.09)	0	0(0-0)	4	3.12(0.85-7.98)	1148	78.96
Chronic Obstructive Pulmonary Disease and Allied Cond	5	2.8(0.91-6.52)	2	1.87(0.23-6.74)	3	2.57(0.53-7.5)	0	0(0-0)	10	2.48^{p}(1.19-4.56)	1148	73.54
Chronic Liver Disease and Cirrhosis	0	0(0-10.81)	0	0(0-16.84)	0	0(0-14.77)	0	0(0-0)	0	0(0-4.55)	1148	
Nephritis, Nephrotic Syndrome and Nephrosis	2	3.69(0.45-13.32)	1	3.14(0.08-17.48)	2	5.68(0.69-20.52)	0	0(0-0)	5	4.12^{p}(1.34-9.62)	1148	80.62
Accidents and Adverse Effects	1	1.07(0.03-5.96)	2	3.52(0.43-12.7)	1	1.55(0.04-8.64)	0	0(0-0)	4	1.86(0.51-4.77)	1148	68.53
Suicide and Self-Inflicted Injury	2	8.08(0.98-29.2)	1	6.36(0.16-35.42)	1	5.64(0.14-31.43)	0	0(0-0)	4	6.87^{p}(1.87-17.6)	1148	66.65
Other Cause of Death	9	1.72(0.79-3.26)	9	2.94^{p}(1.34-5.58)	9	2.62^{p}(1.2-4.97)	0	0(0-0)	27	2.30^{p}(1.52-3.35)	1148	75.47

Table S14: Standardized-mortality ratios (SMRs) for non-cancer causes for cardia during 2015-2019.

Causes	<1 years		1-2 years		2-5 years		>5 years		Total		Total	
	Observed (n)	SMR (95%CI)	Observed (n)	SMR (95%CI)	Observed (n)	Observed (n)	SMR (95%CI)	Observed (n)	SMR (95%CI)	Observed (n)	Patient	Mean Age at Event
Non-GC	259	14.92^{p}(13.16-16.85)	172	13.11^{p}(11.23-15.23)	212	7.74^{p}(6.73-8.85)	90	2.81^{p}(2.26-3.46)	733	8.16^{p}(7.58-8.77)	2696	72.77
GC	155	430.13^{p}(365.08-503.42)	86	318.69^{p}(254.91-393.58)	75	137.72^{p}(108.32-172.63)	23	38.10^{p}(24.15-57.17)	339	190.62^{p}(170.86-212.03)	2696	72.75
All Causes of Death	526	7.61^{p}(6.97-8.29)	324	6.25^{p}(5.59-6.97)	409	3.70^{p}(3.35-4.08)	243	1.71^{p}(1.5-1.94)	1502	4.02^{p}(3.82-4.23)	2696	73.86
Non-cancer causes	112	2.18^{p}(1.79-2.62)	66	1.72^{p}(1.33-2.19)	122	1.48^{p}(1.23-1.76)	130	1.19(0.99-1.41)	430	1.53^{p}(1.39-1.68)	2696	76.62
Septicemia	9	9.27^{p}(4.24-17.6)	0	0(0-5.02)	1	0.63(0.02-3.54)	0	0(0-1.85)	10	1.9(0.91-3.49)	2696	71.56
Other Infectious and Parasitic Diseases including HIV	2	3.37(0.41-12.17)	2	4.45(0.54-16.07)	0	0(0-3.99)	2	1.93(0.23-6.96)	6	2(0.73-4.34)	2696	69.86
Diabetes Mellitus	5	2.32(0.75-5.42)	2	1.23(0.15-4.44)	3	0.87(0.18-2.55)	5	1.18(0.38-2.76)	15	1.31(0.73-2.16)	2696	73.77
Alzheimer's (ICD-9 and 10 only)	0	0(0-1.85)	2	1.34(0.16-4.82)	4	1.15(0.31-2.95)	5	0.87(0.28-2.03)	11	0.86(0.43-1.55)	2696	83.12
Cardiovascular Diseases	39	1.98^{p}(1.41-2.7)	14	0.96(0.53-1.61)	43	1.40^{p}(1.01-1.88)	46	1.16(0.85-1.54)	142	1.35^{p}(1.14-1.6)	2696	78.94
Cerebrovascular Diseases	5	1.43(0.46-3.34)	4	1.55(0.42-3.97)	5	0.91(0.3-2.12)	7	0.94(0.38-1.93)	21	1.1(0.68-1.69)	2696	77.41
Pneumonia and Influenza	1	0.64(0.02-3.54)	2	1.74(0.21-6.28)	1	0.41(0.01-2.3)	3	0.95(0.2-2.77)	7	0.84(0.34-1.74)	2696	71.76
Chronic Obstructive Pulmonary Disease and Allied Cond	5	1.09(0.35-2.55)	12	3.46^{p}(1.79-6.05)	15	2.02^{p}(1.13-3.33)	21	2.24^{p}(1.39-3.43)	53	2.13^{p}(1.6-2.79)	2696	78.22
Chronic Liver Disease and Cirrhosis	2	2.49(0.3-9.01)	3	4.83(1-14.1)	2	1.54(0.19-5.56)	2	1.4(0.17-5.04)	9	2.16(0.99-4.11)	2696	73.67
Nephritis, Nephrotic Syndrome and Nephrosis	2	1.44(0.17-5.2)	1	0.96(0.02-5.37)	6	2.7(0.99-5.88)	3	1.06(0.22-3.09)	12	1.6(0.83-2.8)	2696	70.74
Accidents and Adverse Effects	6	2.90^{p}(1.07-6.32)	1	0.64(0.02-3.56)	5	1.48(0.48-3.46)	4	0.92(0.25-2.36)	16	1.41(0.81-2.29)	2696	71.25
Suicide and Self-Inflicted Injury	3	4.86^{p}(1-14.22)	2	4.24(0.51-15.32)	0	0(0-3.75)	0	0(0-3.4)	5	1.58(0.51-3.7)	2696	68.6
Other Cause of Death	33	2.88^{p}(1.98-4.04)	21	2.42^{p}(1.5-3.71)	37	1.93^{p}(1.36-2.66)	32	1.19(0.82-1.68)	123	1.86^{p}(1.55-2.22)	2696	75.7

Table S15: Standardized-mortality ratios (SMRs) for non-cancer causes for cardia in married patients.

Causes	<1 years		1-2 years		2-5 years		>5 years		Total		Total	
	Observed (n)	SMR (95%CI)	Observed (n)	SMR (95%CI)	Observed (n)	Observed (n)	SMR (95%CI)	Observed (n)	SMR (95%CI)	Observed (n)	Patient	Mean Age at Event
Non-GC	217	24.17[P](21.06-27.61)	105	18.64[P](15.24-22.56)	112	10.82[P](8.91-13.01)	30	3.37[P](2.27-4.81)	464	13.70[P](12.48-15)	1475	73.01
GC	169	924.25[P](790.15-1074.58)	63	550.71[P](423.18-704.59)	45	220.29[P](160.68-294.76)	12	70.73[P](36.55-123.55)	289	430.58[P](382.36-483.19)	1475	76.53
All Causes of Death	481	11.03[P](10.07-12.06)	205	7.73[P](6.71-8.87)	223	4.44[P](3.88-5.07)	107	2.36[P](1.93-2.85)	1016	6.13[P](5.76-6.52)	1475	75.04
Non-cancer causes	95	2.76[P](2.23-3.37)	37	1.78[P](1.25-2.46)	66	1.67[P](1.29-2.12)	65	1.79[P](1.38-2.28)	263	2.01[P](1.77-2.26)	1475	76.99
Septicemia	4	6.46[P](1.76-16.54)	1	2.62(0.07-14.62)	1	1.4(0.04-7.8)	2	3.16(0.38-11.41)	8	3.41[P](1.47-6.72)	1475	72.73
Other Infectious and Parasitic Diseases including HIV	0	0(0-10.69)	2	9.20[P](1.11-33.23)	2	5.01(0.61-18.11)	1	3.08(0.08-17.16)	5	3.89[P](1.26-9.07)	1475	73.63
Diabetes Mellitus	3	2.44(0.5-7.14)	2	2.6(0.32-9.41)	3	2.1(0.43-6.15)	2	1.6(0.19-5.77)	10	2.14[P](1.03-3.94)	1475	74.31
Alzheimer's (ICD-9 and 10 only)	1	0.51(0.01-2.86)	0	0(0-3.25)	5	2.14(0.7-5)	2	0.83(0.1-3.01)	8	1.02(0.44-2.02)	1475	86.45
Cardiovascular Diseases	39	2.99[P](2.13-4.09)	8	1.03(0.45-2.04)	23	1.59[P](1.01-2.38)	21	1.62(1-2.47)	91	1.89[P](1.52-2.32)	1475	80.02
Cerebrovascular Diseases	4	1.53(0.42-3.91)	1	0.65(0.02-3.62)	2	0.69(0.08-2.48)	3	1.12(0.23-3.28)	10	1.03(0.49-1.89)	1475	85.62
Pneumonia and Influenza	4	3.54(0.96-9.06)	2	3.04(0.37-10.97)	5	4.08[P](1.33-9.52)	1	0.94(0.02-5.24)	12	2.94[P](1.52-5.14)	1475	73.62
Chronic Obstructive Pulmonary Disease and Allied Cond	13	4.86[P](2.59-8.31)	4	2.43(0.66-6.21)	2	0.65(0.08-2.33)	5	1.79(0.58-4.18)	24	2.35[P](1.51-3.5)	1475	75.85
Chronic Liver Disease and Cirrhosis	0	0(0-10.22)	1	4.07(0.1-22.69)	1	2.17(0.06-12.12)	0	0(0-9.57)	2	1.38(0.17-4.98)	1475	69.42
Nephritis, Nephrotic Syndrome and Nephrosis	2	2.22(0.27-8.01)	5	9.10[P](2.96-21.25)	3	2.93(0.6-8.56)	4	4.42[P](1.2-11.31)	14	4.14[P](2.26-6.95)	1475	80.01
Accidents and Adverse Effects	2	1.65(0.2-5.95)	3	3.94(0.81-11.53)	3	2.04(0.42-5.97)	7	5.14[P](2.07-10.6)	15	3.12[P](1.75-5.15)	1475	69.33
Suicide and Self-Inflicted Injury	6	23.13[P](8.49-50.35)	0	0(0-21.28)	1	3.09(0.08-17.21)	4	14.69[P](4-37.61)	11	10.69[P](5.34-19.13)	1475	64.95
Other Cause of Death	17	2.10[P](1.22-3.36)	8	1.62(0.7-3.18)	15	1.54(0.86-2.54)	13	1.4(0.75-2.4)	53	1.65[P](1.24-2.16)	1475	75.61

Table S16: Standardized-mortality ratios (SMRs) for non-cancer causes for cardia in unmarried patients.

Causes	<1 years		1-2 years		2-5 years		>5 years		Total		Total	
	Observed (n)	SMR (95%CI)	Observed (n)	SMR (95%CI)	Observed (n)	Observed (n)	SMR (95%CI)	Observed (n)	SMR (95%CI)	Observed (n)	Patient	Mean Age at Event
Non-GC	27	21.72[p](14.31-31.6)	12	14.15[p](7.31-24.71)	12	7.08[p](3.66-12.36)	3	2.59(0.53-7.58)	54	10.92[p](8.21-14.25)	213	74.48
GC	23	877.65[p](556.36-1316.91)	5	290.08[p](94.19-676.94)	8	236.37[p](102.05-465.74)	1	48.17[p](1.22-268.39)	37	377.37[p](265.7-520.15)	213	72.19
All Causes of Death	56	10.32[p](7.79-13.4)	21	5.57[p](3.45-8.52)	28	3.54[p](2.35-5.12)	7	1.34(0.54-2.76)	112	5.02[p](4.13-6.04)	213	75.09
Non-cancer causes	6	1.44(0.53-3.14)	4	1.38(0.38-3.53)	8	1.3(0.56-2.55)	3	0.74(0.15-2.17)	21	1.22(0.75-1.86)	213	81.76
Septicemia	1	12.12(0.31-67.51)	0	0(0-65.65)	1	8.73(0.22-48.65)	0	0(0-49.52)	2	6.1(0.74-22.04)	213	76.87
Other Infectious and Parasitic Diseases including HIV	0	0(0-83.14)	0	0(0-120.71)	0	0(0-61.27)	0	0(0-96.15)	0	0(0-21.26)	213	
Diabetes Mellitus	0	0(0-21.68)	0	0(0-32)	0	0(0-15.68)	1	6.38(0.16-35.55)	1	1.48(0.04-8.22)	213	90.17
Alzheimer's (ICD-9 and 10 only)	1	5.01(0.13-27.93)	0	0(0-25.7)	1	2.88(0.07-16.03)	0	0(0-16.97)	2	2.2(0.27-7.96)	213	88.53
Cardiovascular Diseases	1	0.65(0.02-3.61)	0	0(0-3.45)	2	0.89(0.11-3.21)	2	1.36(0.16-4.91)	5	0.79(0.26-1.84)	213	87.4
Cerebrovascular Diseases	1	3.36(0.08-18.7)	0	0(0-18.17)	0	0(0-8.46)	0	0(0-13.48)	1	0.83(0.02-4.6)	213	85.42
Pneumonia and Influenza	0	0(0-28.69)	0	0(0-41.32)	0	0(0-19.38)	0	0(0-32.31)	0	0(0-7.06)	213	
Chronic Obstructive Pulmonary Disease and Allied Cond	1	2.92(0.07-16.27)	1	4.2(0.11-23.4)	1	2.03(0.05-11.32)	0	0(0-11.03)	3	2.13(0.44-6.23)	213	76.87
Chronic Liver Disease and Cirrhosis	0	0(0-67.93)	0	0(0-93.49)	0	0(0-48.05)	0	0(0-68.11)	0	0(0-16.42)	213	
Nephritis, Nephrotic Syndrome and Nephrosis	0	0(0-31.42)	0	0(0-46.03)	1	5.94(0.15-33.09)	0	0(0-34.96)	1	2.12(0.05-11.82)	213	79
Accidents and Adverse Effects	0	0(0-23.96)	1	9.01(0.23-50.21)	1	4.24(0.11-23.6)	0	0(0-22.55)	2	3.01(0.36-10.87)	213	74.38
Suicide and Self-Inflicted Injury	0	0(0-93.86)	0	0(0-128.4)	0	0(0-66.99)	0	0(0-93.75)	0	0(0-22.71)	213	
Other Cause of Death	1	1.02(0.03-5.66)	2	2.87(0.35-10.37)	1	0.66(0.02-3.69)	0	0(0-3.69)	4	0.95(0.26-2.44)	213	78.8

Table S17: Standardized-mortality ratios (SMRs) for non-cancer causes for cardia in unknown marital status patients.

Causes	<1 years		1-2 years		2-5 years		>5 years		Total		Total	
	Observed (n)	SMR (95%CI)	Observed (n)	SMR (95%CI)	Observed (n)	Observed (n)	SMR (95%CI)	Observed (n)	SMR (95%CI)	Observed (n)	Patient	Mean Age at Event
Non-GC	1	78.11^{p}(1.98-435.19)	0	0(0-307.54)	0	0(0-108.8)	2	28.28^{p}(3.42-102.14)	3	23.18^{p}(4.78-67.74)	96	41.42
GC	6	10,933.79^{p}(4012.51-23798.25)	5	9,378.63^{p}(3045.21-21886.58)	3	2,027.79^{p}(418.18-5926.05)	3	1,086.58^{p}(224.08-3175.44)	17	3,194.11^{p}(1860.69-5114.09)	96	37.62
All Causes of Death	8	79.31^{p}(34.24-156.26)	5	54.87^{p}(17.82-128.06)	4	16.90^{p}(4.61-43.28)	6	15.51^{p}(5.69-33.75)	23	28.20^{p}(17.88-42.31)	96	37.79
Non-cancer causes	1	11.43(0.29-63.66)	0	0(0-46.94)	1	4.97(0.13-27.68)	1	3.19(0.08-17.77)	3	4.41(0.91-12.88)	96	35.09
Septicemia	0	0(0-3746.83)	0	0(0-4056.84)	0	0(0-1507.86)	0	0(0-758.24)	0	0(0-400.73)	96	
Other Infectious and Parasitic Diseases including HIV	0	0(0-1343.87)	0	0(0-1623.04)	1	164.57^{p}(4.17-916.9)	0	0(0-438.83)	1	51.28^{p}(1.3-285.72)	96	34.08
Diabetes Mellitus	0	0(0-1792.65)	0	0(0-1864.9)	0	0(0-630.69)	0	0(0-282.29)	0	0(0-160.72)	96	
Alzheimer's (ICD-9 and 10 only)	0	0(0-634189.12)	0	0(0-604139.55)	0	0(0-161494.19)	0	0(0-42223.12)	0	0(0-30204.23)	96	
Cardiovascular Diseases	1	91.81^{p}(2.32-511.55)	0	0(0-352.43)	0	0(0-116.51)	0	0(0-54.23)	1	8.26(0.21-46.03)	96	36.67
Cerebrovascular Diseases	0	0(0-1864.29)	0	0(0-1952.62)	0	0(0-648.6)	0	0(0-311.38)	0	0(0-172.36)	96	
Pneumonia and Influenza	0	0(0-3321.26)	0	0(0-3587.87)	0	0(0-1300.39)	0	0(0-769.63)	0	0(0-377.62)	96	
Chronic Obstructive Pulmonary Disease and Allied Cond	0	0(0-4927.94)	0	0(0-5134.34)	0	0(0-1745.79)	0	0(0-721.1)	0	0(0-424.22)	96	
Chronic Liver Disease and Cirrhosis	0	0(0-1271.17)	0	0(0-1268.46)	0	0(0-452.94)	0	0(0-221.97)	0	0(0-120.66)	96	
Nephritis, Nephrotic Syndrome and Nephrosis	0	0(0-4844.59)	0	0(0-5195.75)	0	0(0-1768.81)	0	0(0-795.89)	0	0(0-450.31)	96	
Accidents and Adverse Effects	0	0(0-124.2)	0	0(0-142.1)	0	0(0-60.59)	0	0(0-48.25)	0	0(0-19.11)	96	
Suicide and Self-Inflicted Injury	0	0(0-335.75)	0	0(0-381.52)	0	0(0-160.18)	0	0(0-135.27)	0	0(0-51.99)	96	
Other Cause of Death	0	0(0-162.87)	0	0(0-183.75)	0	0(0-73.15)	1	13.83(0.35-77.08)	1	6.04(0.15-33.68)	96	34.5

Table S18: Standardized-mortality ratios (SMRs) for non-cancer causes for non-cardia in patients aged <39 years

Causes	<1 years		1-2 years		2-5 years		>5 years		Total		Total	
	Observed (n)	SMR (95%CI)	Observed (n)	SMR (95%CI)	Observed (n)	Observed (n)	SMR (95%CI)	Observed (n)	SMR (95%CI)	Observed (n)	Patient	Mean Age at Event
Non-GC	11	3.68[p](1.84-6.59)	5	1.85(0.6-4.32)	9	1.28(0.58-2.43)	14	1.07(0.59-1.8)	39	1.51[p](1.08-2.07)	1493	62.12
GC	117	1,456.78[p](1204.8-1745.92)	79	1,087.64[p](861.1-1355.53)	85	455.30[p](363.68-562.98)	31	92.00[p](62.51-130.58)	312	461.13[p](411.37-515.24)	1493	58.12
All Causes of Death	157	15.99[p](13.59-18.7)	98	11.22[p](9.11-13.68)	129	5.73[p](4.79-6.81)	89	2.06[p](1.65-2.53)	473	5.61[p](5.12-6.14)	1493	59.41
Non-cancer causes	29	4.30[p](2.88-6.17)	14	2.35[p](1.28-3.94)	35	2.29[p](1.6-3.19)	44	1.47[p](1.07-1.98)	122	2.11[p](1.75-2.52)	1493	61.82
Septicemia	1	6.67(0.17-37.14)	1	7.41(0.19-41.3)	1	2.8(0.07-15.58)	3	4.19(0.86-12.25)	6	4.42[p](1.62-9.61)	1493	58.54
Other Infectious and Parasitic Diseases including HIV	2	8.38[p](1.01-30.26)	0	0(0-18.74)	2	4.41(0.53-15.94)	0	0(0-5.91)	4	2.64(0.72-6.77)	1493	61.12
Diabetes Mellitus	2	4.85(0.59-17.54)	1	2.69(0.07-15.02)	3	3.09(0.64-9.02)	4	2.06(0.56-5.27)	10	2.70[p](1.3-4.97)	1493	63.39
Alzheimer's (ICD-9 and 10 only)	0	0(0-179.51)	0	0(0-168.12)	0	0(0-50.92)	0	0(0-9.33)	0	0(0-7.23)	1493	
Cardiovascular Diseases	9	3.70[p](1.69-7.02)	1	0.46(0.01-2.58)	9	1.62(0.74-3.08)	12	1.1(0.57-1.93)	31	1.48[p](1-2.09)	1493	63.01
Cerebrovascular Diseases	1	2.56(0.06-14.24)	0	0(0-10.5)	3	3.26(0.67-9.52)	2	1(0.12-3.63)	6	1.64(0.6-3.57)	1493	59.64
Pneumonia and Influenza	1	7.64(0.19-42.58)	1	8.34(0.21-46.46)	1	3.12(0.08-17.4)	0	0(0-5.19)	3	2.34(0.48-6.84)	1493	59.41
Chronic Obstructive Pulmonary Disease and Allied Cond	0	0(0-10.18)	1	2.9(0.07-16.16)	3	3.09(0.64-9.04)	3	1.32(0.27-3.86)	7	1.77(0.71-3.65)	1493	61.85
Chronic Liver Disease and Cirrhosis	4	12.47[p](3.4-31.94)	1	3.66(0.09-20.37)	6	9.09[p](3.34-19.79)	2	2.1(0.25-7.57)	13	5.89[p](3.13-10.07)	1493	61.54
Nephritis, Nephrotic Syndrome and Nephrosis	0	0(0-21.89)	1	6.54(0.17-36.42)	1	2.43(0.06-13.53)	3	3.33(0.69-9.72)	5	3.06(0.99-7.14)	1493	60.34
Accidents and Adverse Effects	1	1.7(0.04-9.5)	0	0(0-7.56)	3	2.63(0.54-7.68)	4	2.34(0.64-5.98)	8	2.04(0.88-4.01)	1493	60.58
Suicide and Self-Inflicted Injury	1	4.75(0.12-26.45)	0	0(0-20.9)	0	0(0-9.24)	0	0(0-7.17)	1	0.77(0.02-4.28)	1493	65
Other Cause of Death	7	5.29[p](2.13-10.9)	7	5.98[p](2.4-12.32)	3	0.99(0.2-2.89)	11	1.75(0.87-3.13)	28	2.37[p](1.57-3.42)	1493	62.1

Table S19: Standardized-mortality ratios (SMRs) for non-cancer causes for non-cardia in patients aged 40-65 years.

Causes	<1 years		1-2 years		2-5 years		>5 years		Total		Total	
	Observed (n)	SMR (95%CI)	Observed (n)	SMR (95%CI)	Observed (n)	Observed (n)	SMR (95%CI)	Observed (n)	SMR (95%CI)	Observed (n)	Patient	Mean Age at Event
Non-GC	48	1.60[p](1.18-2.12)	35	1.52[p](1.06-2.12)	49	0.96(0.71-1.27)	68	1.18(0.91-1.49)	200	1.24[p](1.07-1.42)	3748	81.81
GC	657	774.75[p](716.64-836.33)	217	330.63[p](288.1-377.67)	233	162.06[p](141.92-184.26)	54	34.20[p](25.69-44.63)	1161	256.81[p](242.25-272.01)	3748	82.19
All Causes of Death	937	6.10[p](5.72-6.5)	344	2.91[p](2.61-3.23)	496	1.83[p](1.67-2)	478	1.38[p](1.26-1.51)	2255	2.54[p](2.43-2.64)	3748	83.22
Non-cancer causes	232	1.89[p](1.66-2.15)	92	0.97(0.78-1.19)	214	0.98(0.85-1.12)	356	1.24[p](1.12-1.38)	894	1.24[p](1.16-1.32)	3748	84.87
Septicemia	10	4.37[p](2.09-8.03)	0	0(0-2.11)	4	1.01(0.28-2.59)	8	1.67(0.72-3.29)	22	1.72[p](1.08-2.61)	3748	81.94
Other Infectious and Parasitic Diseases including HIV	4	3.56(0.97-9.11)	2	2.29(0.28-8.27)	6	3.07[p](1.13-6.68)	3	1.35(0.28-3.95)	15	2.43[p](1.36-4.01)	3748	81.69
Diabetes Mellitus	10	2.08(1-3.83)	5	1.36(0.44-3.17)	6	0.73(0.27-1.58)	9	0.91(0.42-1.73)	30	1.13(0.76-1.61)	3748	82.55
Alzheimer's (ICD-9 and 10 only)	5	0.69(0.22-1.62)	2	0.35(0.04-1.26)	4	0.28[p](0.08-0.72)	23	1.06(0.67-1.59)	34	0.70[p](0.48-0.97)	3748	90.38
Cardiovascular Diseases	91	1.96[p](1.58-2.4)	34	0.96(0.66-1.34)	71	0.89(0.69-1.12)	147	1.44[p](1.21-1.69)	343	1.30[p](1.16-1.44)	3748	85.57
Cerebrovascular Diseases	18	1.74[p](1.03-2.74)	6	0.75(0.28-1.64)	21	1.16(0.72-1.77)	29	1.22(0.82-1.76)	74	1.23(0.97-1.54)	3748	85.01
Pneumonia and Influenza	9	2.01(0.92-3.81)	4	1.16(0.32-2.97)	14	1.79(0.98-3.01)	13	1.3(0.69-2.22)	40	1.55[p](1.11-2.12)	3748	85.12
Chronic Obstructive Pulmonary Disease and Allied Cond	20	2.26[p](1.38-3.49)	7	1.02(0.41-2.11)	19	1.22(0.73-1.9)	15	0.78(0.44-1.29)	61	1.21(0.92-1.55)	3748	81.11
Chronic Liver Disease and Cirrhosis	2	2.57(0.31-9.28)	1	1.68(0.04-9.35)	5	3.89[p](1.26-9.08)	3	2.21(0.45-6.45)	11	2.74[p](1.37-4.9)	3748	76.15
Nephritis, Nephrotic Syndrome and Nephrosis	9	2.53[p](1.16-4.8)	4	1.46(0.4-3.75)	8	1.28(0.55-2.53)	11	1.42(0.71-2.55)	32	1.58[p](1.08-2.23)	3748	86.22
Accidents and Adverse Effects	4	1.11(0.3-2.83)	4	1.42(0.39-3.63)	7	1.06(0.42-2.17)	15	1.67(0.94-2.76)	30	1.36(0.92-1.94)	3748	82.76

Suicide and Self-Inflicted Injury	1	2.16(0.05-12.05)	0	0(0-10.41)	1	1.29(0.03-7.16)	0	0(0-4.07)	2	0.8(0.1-2.89)	3748	78.88
Other Cause of Death	49	1.71[p](1.27-2.26)	23	1.02(0.65-1.53)	48	0.89(0.66-1.18)	80	1.09(0.86-1.35)	200	1.12(0.97-1.29)	3748	85.34

Table S20: Standardized-mortality ratios (SMRs) for non-cancer causes for non-cardia in patients aged >65 years.

Causes	<1 years		1-2 years		2-5 years		>5 years		Total		Total	
	Observed (n)	SMR (95%CI)	Observed (n)	SMR (95%CI)	Observed (n)	Observed (n)	SMR (95%CI)	Observed (n)	SMR (95%CI)	Observed (n)	Patient	Mean Age at Event
Non-GC	32	1.56[p](1.07-2.21)	23	1.45(0.92-2.17)	33	0.92(0.64-1.3)	54	1.19(0.89-1.55)	142	1.21[p](1.02-1.42)	2889	76.81
GC	424	687.23[p](623.36-755.86)	168	347.34[p](296.8-404.02)	171	159.43[p](136.43-185.2)	51	39.65[p](29.52-52.14)	814	235.30[p](219.41-252.04)	2889	75.49
All Causes of Death	598	6.57[p](6.05-7.12)	255	3.65[p](3.21-4.12)	347	2.16[p](1.94-2.4)	305	1.39[p](1.24-1.55)	1505	2.78[p](2.64-2.92)	2889	77.56
Non-cancer causes	142	2.03[p](1.71-2.39)	64	1.19(0.92-1.53)	143	1.15(0.97-1.36)	200	1.15[p](1-1.33)	549	1.30[p](1.2-1.42)	2889	80.82
Septicemia	4	3.02(0.82-7.74)	0	0(0-3.65)	4	1.73(0.47-4.42)	5	1.6(0.52-3.74)	13	1.67(0.89-2.86)	2889	76.62
Other Infectious and Parasitic Diseases including HIV	3	3.68(0.76-10.75)	2	3.16(0.38-11.42)	6	4.25[p](1.56-9.24)	3	1.74(0.36-5.09)	14	3.06[p](1.67-5.13)	2889	75.05
Diabetes Mellitus	4	1.35(0.37-3.45)	5	2.16(0.7-5.05)	7	1.32(0.53-2.73)	11	1.55(0.77-2.78)	27	1.53[p](1.01-2.22)	2889	78.45
Alzheimer's (ICD-9 and 10 only)	2	0.7(0.08-2.54)	0	0(0-1.69)	2	0.37(0.05-1.34)	10	1.14(0.54-2.09)	14	0.73(0.4-1.22)	2889	88.65
Cardiovascular Diseases	61	2.22[p](1.7-2.85)	19	0.91(0.55-1.42)	46	0.97(0.71-1.29)	79	1.22(0.96-1.52)	205	1.28[p](1.11-1.46)	2889	81.87
Cerebrovascular Diseases	13	2.50[p](1.33-4.28)	5	1.27(0.41-2.95)	9	1(0.46-1.89)	12	0.95(0.49-1.66)	39	1.27(0.9-1.73)	2889	82.09
Pneumonia and Influenza	2	0.77(0.09-2.79)	3	1.52(0.31-4.44)	9	2(0.91-3.8)	7	1.14(0.46-2.36)	21	1.38(0.86-2.11)	2889	83.21
Chronic Obstructive Pulmonary Disease and Allied Cond	13	2.43[p](1.29-4.16)	4	0.97(0.26-2.48)	14	1.48(0.81-2.48)	11	0.86(0.43-1.54)	42	1.32(0.95-1.79)	2889	78.82
Chronic Liver Disease and Cirrhosis	5	6.73[p](2.19-15.71)	1	1.7(0.04-9.47)	7	5.36[p](2.16-11.05)	3	1.86(0.38-5.45)	16	3.77[p](2.15-6.12)	2889	66.26
Nephritis, Nephrotic Syndrome and Nephrosis	4	1.87(0.51-4.78)	4	2.44(0.66-6.25)	7	1.86(0.75-3.83)	2	0.39(0.05-1.41)	17	1.34(0.78-2.15)	2889	84.35

Accidents and Adverse Effects	2	0.76(0.09-2.75)	4	1.96(0.53-5.02)	5	1.05(0.34-2.46)	9	1.36(0.62-2.58)	20	1.25(0.76-1.93)	2889	77.24
Suicide and Self-Inflicted Injury	2	3.43(0.42-12.39)	0	0(0-8.01)	1	0.97(0.02-5.43)	0	0(0-2.91)	3	0.9(0.19-2.63)	2889	74.25
Other Cause of Death	27	1.77^{p}(1.16-2.57)	17	1.43(0.84-2.3)	26	0.92(0.6-1.35)	48	1.16(0.85-1.53)	118	1.22^{p}(1.01-1.46)	2889	81.85

Table S21: Standardized-mortality ratios (SMRs) for non-cancer causes for non-cardia in male patients

Causes	<1 years		1-2 years		2-5 years		>5 years		Total		Total	
	Observed (n)	SMR (95%CI)	Observed (n)	SMR (95%CI)	Observed (n)	Observed (n)	SMR (95%CI)	Observed (n)	SMR (95%CI)	Observed (n)	Patient	Mean Age at Event
Non-GC	28	2.23^{p}(1.48-3.22)	17	1.73^{p}(1.01-2.76)	25	1.12(0.73-1.66)	30	1.18(0.8-1.69)	100	1.43^{p}(1.16-1.74)	2448	80.03
GC	356	1,141.38^{p}(1025.89-1266.33)	133	541.06^{p}(453.02-641.22)	150	271.10^{p}(229.45-318.12)	37	58.50^{p}(41.19-80.64)	676	387.74^{p}(359.05-418.1)	2448	78.03
All Causes of Death	504	6.95^{p}(6.36-7.59)	192	3.35^{p}(2.9-3.86)	282	2.12^{p}(1.88-2.38)	268	1.58^{p}(1.4-1.78)	1246	2.88^{p}(2.72-3.05)	2448	80.18
Non-cancer causes	120	2.01^{p}(1.67-2.41)	42	0.89(0.64-1.2)	107	0.97(0.79-1.17)	201	1.40^{p}(1.21-1.61)	470	1.30^{p}(1.19-1.43)	2448	83.31
Septicemia	7	6.27^{p}(2.52-12.91)	1	1.15(0.03-6.39)	1	0.5(0.01-2.8)	6	2.51(0.92-5.47)	15	2.36^{p}(1.32-3.89)	2448	77.18
Other Infectious and Parasitic Diseases including HIV	3	5.45^{p}(1.12-15.93)	0	0(0-8.38)	3	2.99(0.62-8.75)	0	0(0-3.26)	6	1.92(0.71-4.18)	2448	75.54
Diabetes Mellitus	8	3.56^{p}(1.54-7.01)	1	0.57(0.01-3.19)	2	0.51(0.06-1.82)	2	0.42(0.05-1.52)	13	1.02(0.54-1.75)	2448	76.32
Alzheimer's (ICD-9 and 10 only)	3	0.68(0.14-2)	2	0.56(0.07-2.03)	2	0.23^{p}(0.03-0.82)	13	0.97(0.52-1.66)	20	0.66(0.41-1.02)	2448	91.6
Cardiovascular Diseases	40	1.86^{p}(1.33-2.54)	16	0.95(0.55-1.55)	34	0.89(0.61-1.24)	80	1.65^{p}(1.31-2.05)	170	1.36^{p}(1.16-1.58)	2448	85.63
Cerebrovascular Diseases	6	1.08(0.4-2.35)	1	0.23(0.01-1.28)	15	1.49(0.83-2.46)	19	1.45(0.87-2.26)	41	1.24(0.89-1.68)	2448	84.07
Pneumonia and Influenza	8	3.94^{p}(1.7-7.77)	2	1.26(0.15-4.54)	6	1.65(0.61-3.6)	6	1.31(0.48-2.84)	22	1.86^{p}(1.16-2.81)	2448	83.44
Chronic Obstructive Pulmonary Disease and Allied Cond	7	1.81(0.73-3.74)	4	1.3(0.35-3.34)	8	1.12(0.49-2.22)	7	0.81(0.33-1.67)	26	1.15(0.75-1.68)	2448	79.61
Chronic Liver Disease and Cirrhosis	1	2.78(0.07-15.5)	1	3.52(0.09-19.59)	4	6.18^{p}(1.68-15.81)	2	2.77(0.34-10.01)	8	3.97^{p}(1.72-7.83)	2448	72.2
Nephritis, Nephrotic Syndrome and Nephrosis	5	3.15^{p}(1.02-7.35)	1	0.8(0.02-4.47)	2	0.7(0.08-2.51)	12	3.41^{p}(1.76-5.96)	20	2.17^{p}(1.32-3.35)	2448	81.34

Accidents and Adverse Effects	3	1.87(0.38-5.45)	0	0(0-2.84)	5	1.61(0.52-3.77)	10	2.43^{P}(1.16-4.46)	18	1.78^{P}(1.05-2.81)	2448	79.04
Suicide and Self-Inflicted Injury	0	0(0-36.59)	0	0(0-46.25)	0	0(0-21.28)	0	0(0-20.49)	0	0(0-6.91)	2448	
Other Cause of Death	29	1.97^{P}(1.32-2.83)	13	1.1(0.59-1.88)	25	0.87(0.57-1.29)	44	1.15(0.83-1.54)	111	1.19(0.98-1.43)	2448	82.73

Table S22: Standardized-mortality ratios (SMRs) for non-cancer causes for non-cardia in female patients.

Causes	<1 years		1-2 years		2-5 years		>5 years		Total		Total	
	Observed (n)	SMR (95%CI)	Observed (n)	SMR (95%CI)	Observed (n)	Observed (n)	SMR (95%CI)	Observed (n)	SMR (95%CI)	Observed (n)	Patient	Mean Age at Event
Non-GC	42	1.99^{P}(1.43-2.69)	28	1.72^{P}(1.14-2.48)	37	1.02(0.72-1.41)	46	1.04(0.76-1.39)	153	1.30^{P}(1.1-1.52)	3044	78.77
GC	499	1,351.03^{P}(1235.08-1474.94)	192	680.36^{P}(587.53-783.71)	191	311.52^{P}(268.9-358.97)	48	66.24^{P}(48.84-87.83)	930	467.51^{P}(437.94-498.55)	3044	76.92
All Causes of Death	711	6.63^{P}(6.15-7.14)	288	3.47^{P}(3.08-3.89)	355	1.87^{P}(1.68-2.07)	339	1.34^{P}(1.2-1.49)	1693	2.67^{P}(2.55-2.8)	3044	79.03
Non-cancer causes	170	1.98^{P}(1.7-2.3)	68	1.02(0.79-1.3)	127	0.83^{P}(0.69-0.99)	245	1.18^{P}(1.04-1.34)	610	1.19^{P}(1.1-1.29)	3044	82.3
Septicemia	6	4.09^{P}(1.5-8.9)	1	0.88(0.02-4.89)	3	1.16(0.24-3.38)	8	2.37^{P}(1.02-4.66)	18	2.10^{P}(1.24-3.31)	3044	78.59
Other Infectious and Parasitic Diseases including HIV	6	7.92^{P}(2.91-17.24)	1	1.68(0.04-9.37)	5	3.77^{P}(1.22-8.8)	1	0.64(0.02-3.55)	13	3.06^{P}(1.63-5.23)	3044	74.26
Diabetes Mellitus	9	3.32^{P}(1.52-6.3)	2	0.96(0.12-3.46)	1	0.21(0.01-1.19)	8	1.35(0.58-2.65)	20	1.3(0.79-2)	3044	78.05
Alzheimer's (ICD-9 and 10 only)	2	0.38(0.05-1.36)	1	0.24(0.01-1.32)	2	0.19^{P}(0.02-0.7)	11	0.7(0.35-1.26)	16	0.45^{P}(0.26-0.73)	3044	91.7
Cardiovascular Diseases	68	2.10^{P}(1.63-2.67)	22	0.89(0.56-1.35)	43	0.77(0.56-1.04)	96	1.29^{P}(1.04-1.57)	229	1.22^{P}(1.07-1.39)	3044	85.03
Cerebrovascular Diseases	15	2.29^{P}(1.28-3.77)	3	0.6(0.12-1.74)	14	1.22(0.67-2.05)	25	1.62^{P}(1.05-2.39)	57	1.48^{P}(1.12-1.92)	3044	83.21
Pneumonia and Influenza	5	1.77(0.58-4.14)	2	0.93(0.11-3.37)	5	1.05(0.34-2.46)	4	0.64(0.18-1.65)	16	1.01(0.57-1.63)	3044	81.79
Chronic Obstructive Pulmonary Disease and Allied Cond	14	2.03^{P}(1.11-3.4)	7	1.3(0.52-2.69)	15	1.22(0.68-2.01)	14	0.89(0.49-1.49)	50	1.24(0.92-1.64)	3044	78.06
Chronic Liver Disease and Cirrhosis	5	6.99^{P}(2.27-16.32)	2	3.55(0.43-12.82)	6	4.82^{P}(1.77-10.5)	5	3.41^{P}(1.11-7.95)	18	4.51^{P}(2.67-7.13)	3044	69.49

Nephritis, Nephrotic Syndrome and Nephrosis	5	2.31(0.75-5.4)	4	2.39(0.65-6.13)	5	1.31(0.43-3.07)	8	1.62(0.7-3.18)	22	1.75^{p}(1.1-2.65)	3044	82.8
Accidents and Adverse Effects	3	1.04(0.21-3.04)	4	1.77(0.48-4.52)	7	1.33(0.53-2.73)	13	1.79(0.95-3.05)	27	1.52^{p}(1-2.22)	3044	78.63
Suicide and Self-Inflicted Injury	0	0(0-7.07)	0	0(0-9.04)	1	1.11(0.03-6.2)	0	0(0-3.36)	1	0.34(0.01-1.91)	3044	70.51
Other Cause of Death	32	1.56^{p}(1.06-2.2)	19	1.17(0.7-1.83)	20	0.52^{p}(0.32-0.8)	52	0.95(0.71-1.25)	123	0.95(0.79-1.13)	3044	82.13

Table S23: Standardized-mortality ratios (SMRs) for non-cancer causes for non-cardia in white patients.

Causes	<1 years		1-2 years		2-5 years		>5 years		Total		Total	
	Observed (n)	SMR (95%CI)	Observed (n)	SMR (95%CI)	Observed (n)	Observed (n)	SMR (95%CI)	Observed (n)	SMR (95%CI)	Observed (n)	Patient	Mean Age at Event
Non-GC	14	2.66^{p}(1.45-4.46)	6	1.6(0.59-3.48)	9	1.08(0.49-2.05)	17	1.71(1-2.74)	46	1.69^{p}(1.24-2.25)	814	74.99
GC	148	842.19^{p}(711.98-989.33)	57	455.37^{p}(344.89-589.98)	39	143.04^{p}(101.72-195.54)	16	50.71^{p}(28.99-82.35)	260	292.44^{p}(257.97-330.23)	814	73.75
All Causes of Death	211	8.57^{p}(7.45-9.81)	78	4.48^{p}(3.54-5.59)	98	2.50^{p}(2.03-3.05)	82	1.67^{p}(1.33-2.07)	469	3.60^{p}(3.28-3.94)	814	75.07
Non-cancer causes	49	2.55^{p}(1.89-3.38)	15	1.11(0.62-1.83)	50	1.64^{p}(1.21-2.16)	49	1.26(0.93-1.67)	163	1.60^{p}(1.36-1.86)	814	77.2
Septicemia	3	5.38^{p}(1.11-15.72)	0	0(0-9.34)	1	1.15(0.03-6.39)	3	2.87(0.59-8.38)	7	2.44(0.98-5.02)	814	71.44
Other Infectious and Parasitic Diseases including HIV	0	0(0-13.51)	0	0(0-18.65)	1	2.35(0.06-13.11)	0	0(0-7.87)	1	0.73(0.02-4.08)	814	34.08
Diabetes Mellitus	2	1.79(0.22-6.46)	2	2.52(0.31-9.11)	4	2.27(0.62-5.82)	1	0.46(0.01-2.57)	9	1.54(0.71-2.93)	814	77.67
Alzheimer's (ICD-9 and 10 only)	3	3.49(0.72-10.21)	0	0(0-6.07)	0	0(0-2.5)	4	1.83(0.5-4.7)	7	1.37(0.55-2.82)	814	88.01
Cardiovascular Diseases	20	2.64^{p}(1.61-4.08)	6	1.13(0.42-2.47)	17	1.45(0.84-2.32)	22	1.51(0.95-2.29)	65	1.66^{p}(1.28-2.12)	814	76.51
Cerebrovascular Diseases	1	0.6(0.02-3.33)	2	1.71(0.21-6.16)	4	1.52(0.41-3.88)	3	0.89(0.18-2.6)	10	1.13(0.54-2.08)	814	80.89
Pneumonia and Influenza	2	3.63(0.44-13.1)	1	2.62(0.07-14.59)	2	2.38(0.29-8.6)	1	0.98(0.02-5.47)	6	2.15(0.79-4.68)	814	74.92
Chronic Obstructive Pulmonary Disease and Allied Cond	6	6.63^{p}(2.43-14.42)	0	0(0-5.69)	3	2.02(0.42-5.91)	2	1.05(0.13-3.81)	11	2.23^{p}(1.11-3.99)	814	81.74

Chronic Liver Disease and Cirrhosis	1	7.41(0.19-41.28)	0	0(0-37.8)	2	9.26[p](1.12-33.47)	0	0(0-14.18)	3	4.23(0.87-12.37)	814	60.36
Nephritis, Nephrotic Syndrome and Nephrosis	1	1.21(0.03-6.76)	0	0(0-6.33)	2	1.53(0.19-5.53)	1	0.62(0.02-3.46)	4	0.93(0.25-2.37)	814	71.09
Accidents and Adverse Effects	1	2.08(0.05-11.6)	0	0(0-10.76)	2	2.59(0.31-9.36)	1	1(0.03-5.58)	4	1.54(0.42-3.95)	814	73.17
Suicide and Self-Inflicted Injury	0	0(0-101.62)	0	0(0-141.69)	0	0(0-64.8)	0	0(0-57.13)	0	0(0-20.07)	814	
Other Cause of Death	9	2.14(0.98-4.07)	4	1.34(0.36-3.43)	12	1.72(0.89-3.01)	11	1.19(0.59-2.13)	36	1.54[p](1.08-2.13)	814	79.05

Table S24: Standardized-mortality ratios (SMRs) for non-cancer causes for non-cardia in black patients.

Causes	<1 years		1-2 years		2-5 years		>5 years		Total		Total	
	Observed (n)	SMR (95%CI)	Observed (n)	SMR (95%CI)	Observed (n)	Observed (n)	SMR (95%CI)	Observed (n)	SMR (95%CI)	Observed (n)	Patient	Mean Age at Event
Non-GC	0	0(0-43.41)	0	0(0-63.98)	0	0(0-22.56)	0	0(0-30.19)	0	0(0-8.61)	29	
GC	5	1,032.24[p](335.17-2408.92)	3	983.24[p](202.77-2873.45)	1	110.69[p](2.8-616.74)	3	457.51[p](94.35-1337.04)	12	510.94[p](264.01-892.5)	29	71.05
All Causes of Death	7	18.70[p](7.52-38.52)	4	18.03[p](4.91-46.17)	1	1.25(0.03-6.99)	3	4.88[p](1.01-14.27)	15	7.47[p](4.18-12.32)	29	71.77
Non-cancer causes	2	7.03(0.85-25.39)	1	6.21(0.16-34.58)	0	0(0-5.9)	0	0(0-7.6)	3	1.93(0.4-5.63)	29	74.67
Septicemia	0	0(0-752.38)	0	0(0-1262.52)	0	0(0-350.79)	0	0(0-485.11)	0	0(0-142.18)	29	
Other Infectious and Parasitic Diseases including HIV	0	0(0-889.71)	0	0(0-1328.89)	0	0(0-487.58)	0	0(0-687.34)	0	0(0-185.79)	29	
Diabetes Mellitus	0	0(0-210.26)	0	0(0-347.36)	0	0(0-105.36)	0	0(0-137.29)	0	0(0-40.97)	29	
Alzheimer's (ICD-9 and 10 only)	0	0(0-357.8)	0	0(0-692.07)	0	0(0-116.08)	0	0(0-118.72)	0	0(0-47)	29	
Cardiovascular Diseases	0	0(0-34.6)	0	0(0-64.13)	0	0(0-16.67)	0	0(0-21.79)	0	0(0-6.65)	29	
Cerebrovascular Diseases	0	0(0-119.55)	0	0(0-222.21)	0	0(0-59.62)	0	0(0-75.78)	0	0(0-23.35)	29	
Pneumonia and Influenza	0	0(0-277.03)	0	0(0-514.79)	0	0(0-121.07)	0	0(0-170.96)	0	0(0-50.86)	29	
Chronic Obstructive Pulmonary Disease and Allied Cond	0	0(0-253.4)	0	0(0-389.25)	0	0(0-111.13)	0	0(0-157.36)	0	0(0-45.73)	29	

Chronic Liver Disease and Cirrhosis	0	0(0-894.06)	0	0(0-1454.26)	0	0(0-635.2)	0	0(0-813.03)	0	0(0-216.9)	29	
Nephritis, Nephrotic Syndrome and Nephrosis	1	116.80^{p}(2.96-650.76)	0	0(0-684.64)	0	0(0-197.25)	0	0(0-262.11)	1	21.4(0.54-119.24)	29	83.51
Accidents and Adverse Effects	0	0(0-347.11)	0	0(0-577.09)	0	0(0-166.17)	0	0(0-218.35)	0	0(0-65.74)	29	
Suicide and Self-Inflicted Injury	0	0(0-1835.36)	0	0(0-2993.22)	0	0(0-1323.28)	0	0(0-1656.11)	0	0(0-446.74)	29	
Other Cause of Death	1	17.54(0.44-97.74)	1	30.17(0.76-168.1)	0	0(0-25.69)	0	0(0-32.39)	2	5.75(0.7-20.78)	29	70.25

Table S25: Standardized-mortality ratios (SMRs) for non-cancer causes for non-cardia in American Indian/Alaska Native patients.

Causes	<1 years		1-2 years		2-5 years		>5 years		Total		Total	
	Observed (n)	SMR (95%CI)	Observed (n)	SMR (95%CI)	Observed (n)	Observed (n)	SMR (95%CI)	Observed (n)	SMR (95%CI)	Observed (n)	Patient	Mean Age at Event
Non-GC	4	0.61(0.17-1.56)	6	1.07(0.39-2.34)	12	0.91(0.47-1.59)	21	1.27(0.79-1.94)	43	1.03(0.74-1.38)	1450	79.23
GC	128	337.77^{p}(281.8-401.62)	49	153.58^{p}(113.62-203.04)	90	123.11^{p}(98.99-151.32)	21	24.08^{p}(14.91-36.82)	288	125.16^{p}(111.12-140.48)	1450	78.58
All Causes of Death	173	5.53^{p}(4.73-6.41)	77	2.91^{p}(2.3-3.64)	175	2.75^{p}(2.36-3.19)	149	1.71^{p}(1.45-2.01)	574	2.75^{p}(2.53-2.99)	1450	81.1
Non-cancer causes	41	1.68^{p}(1.21-2.28)	22	1.07(0.67-1.62)	73	1.47^{p}(1.15-1.84)	107	1.54^{p}(1.26-1.86)	243	1.48^{p}(1.3-1.68)	1450	84.41
Septicemia	2	4.87(0.59-17.58)	0	0(0-10.64)	1	1.21(0.03-6.73)	0	0(0-3.42)	3	1.13(0.23-3.29)	1450	79.7
Other Infectious and Parasitic Diseases including HIV	0	0(0-11.15)	1	3.6(0.09-20.04)	3	4.57(0.94-13.36)	2	2.47(0.3-8.94)	6	2.89^{p}(1.06-6.3)	1450	84.07
Diabetes Mellitus	1	0.73(0.02-4.07)	2	1.72(0.21-6.2)	4	1.44(0.39-3.68)	4	1.08(0.29-2.77)	11	1.22(0.61-2.18)	1450	77.29
Alzheimer's (ICD-9 and 10 only)	0	0(0-3.52)	1	1.11(0.03-6.21)	2	0.84(0.1-3.04)	8	1.87(0.81-3.68)	11	1.28(0.64-2.29)	1450	89.98
Cardiovascular Diseases	13	1.45(0.77-2.48)	7	0.93(0.38-1.92)	20	1.12(0.68-1.73)	41	1.69^{p}(1.22-2.3)	81	1.38^{p}(1.1-1.72)	1450	85.12
Cerebrovascular Diseases	3	1.2(0.25-3.51)	1	0.48(0.01-2.68)	6	1.22(0.45-2.64)	3	0.44(0.09-1.28)	13	0.79(0.42-1.36)	1450	84.35
Pneumonia and Influenza	3	2.43(0.5-7.09)	2	1.93(0.23-6.98)	8	3.19^{p}(1.38-6.28)	8	2.31(1-4.55)	21	2.55^{p}(1.58-3.89)	1450	86.9
Chronic Obstructive Pulmonary Disease and Allied Cond	0	0(0-2.67)	1	0.86(0.02-4.78)	4	1.43(0.39-3.65)	2	0.53(0.06-1.93)	7	0.77(0.31-1.59)	1450	82.59

Chronic Liver Disease and Cirrhosis	0	0(0-14.89)	0	0(0-17.64)	3	6.15[p](1.27-17.99)	0	0(0-6.16)	3	1.94(0.4-5.68)	1450	68.58
Nephritis, Nephrotic Syndrome and Nephrosis	2	2.72(0.33-9.82)	1	1.6(0.04-8.9)	2	1.32(0.16-4.76)	5	2.43(0.79-5.68)	10	2.03(0.97-3.73)	1450	87.13
Accidents and Adverse Effects	1	1.17(0.03-6.53)	0	0(0-5.08)	1	0.57(0.01-3.16)	5	2.03(0.66-4.75)	7	1.21(0.49-2.49)	1450	78.83
Suicide and Self-Inflicted Injury	2	16.12[p](1.95-58.24)	0	0(0-35.01)	0	0(0-15.24)	0	0(0-12.99)	2	2.65(0.32-9.56)	1450	76.13
Other Cause of Death	14	2.72[p](1.49-4.56)	6	1.36(0.5-2.96)	19	1.73[p](1.04-2.7)	29	1.80[p](1.2-2.58)	68	1.85[p](1.44-2.35)	1450	84.59

Table S26: Standardized-mortality ratios (SMRs) for non-cancer causes for non-cardia in Asian/Pacific Islander patients.

Causes	<1 years		1-2 years		2-5 years		>5 years		Total		Total	
	Observed (n)	SMR (95%CI)	Observed (n)	SMR (95%CI)	Observed (n)	Observed (n)	SMR (95%CI)	Observed (n)	SMR (95%CI)	Observed (n)	Patie nt	Mean Age at Event
Non-GC	0	0(0-0)	0	0(0-0)	0	0(0-0)	0	0(0-0)	0	0(0-0)	0	
GC	0	0(0-0)	0	0(0-0)	0	0(0-0)	0	0(0-0)	0	0(0-0)	0	
All Causes of Death	0	0(0-0)	0	0(0-0)	0	0(0-0)	0	0(0-0)	0	0(0-0)	0	
Non-cancer causes	0	0(0-0)	0	0(0-0)	0	0(0-0)	0	0(0-0)	0	0(0-0)	0	
Septicemia	0	0(0-0)	0	0(0-0)	0	0(0-0)	0	0(0-0)	0	0(0-0)	0	
Other Infectious and Parasitic Diseases including HIV	0	0(0-0)	0	0(0-0)	0	0(0-0)	0	0(0-0)	0	0(0-0)	0	
Diabetes Mellitus	0	0(0-0)	0	0(0-0)	0	0(0-0)	0	0(0-0)	0	0(0-0)	0	
Alzheimer's (ICD-9 and 10 only)	0	0(0-0)	0	0(0-0)	0	0(0-0)	0	0(0-0)	0	0(0-0)	0	
Cardiovascular Diseases	0	0(0-0)	0	0(0-0)	0	0(0-0)	0	0(0-0)	0	0(0-0)	0	
Cerebrovascular Diseases	0	0(0-0)	0	0(0-0)	0	0(0-0)	0	0(0-0)	0	0(0-0)	0	
Pneumonia and Influenza	0	0(0-0)	0	0(0-0)	0	0(0-0)	0	0(0-0)	0	0(0-0)	0	
Chronic Obstructive Pulmonary Disease and Allied Cond	0	0(0-0)	0	0(0-0)	0	0(0-0)	0	0(0-0)	0	0(0-0)	0	

Chronic Liver Disease and Cirrhosis	0	0(0-0)	0	0(0-0)	0	0(0-0)	0	0(0-0)	0	0(0-0)	0	
Nephritis, Nephrotic Syndrome and Nephrosis	0	0(0-0)	0	0(0-0)	0	0(0-0)	0	0(0-0)	0	0(0-0)	0	
Accidents and Adverse Effects	0	0(0-0)	0	0(0-0)	0	0(0-0)	0	0(0-0)	0	0(0-0)	0	
Suicide and Self-Inflicted Injury	0	0(0-0)	0	0(0-0)	0	0(0-0)	0	0(0-0)	0	0(0-0)	0	
Other Cause of Death	0	0(0-0)	0	0(0-0)	0	0(0-0)	0	0(0-0)	0	0(0-0)	0	

Table S27: Standardized-mortality ratios (SMRs) for non-cancer causes for non-cardia in unknown race patients.

Causes	<1 years		1-2 years		2-5 years		>5 years		Total		Total	
	Observed (n)	SMR (95%CI)	Observed (n)	SMR (95%CI)	Observed (n)	Observed (n)	SMR (95%CI)	Observed (n)	SMR (95%CI)	Observed (n)	Patient	Mean Age at Event
Non-GC	3	1.24(0.26-3.63)	2	0.96(0.12-3.48)	3	0.6(0.12-1.75)	13	1.13(0.6-1.94)	21	1(0.62-1.53)	345	79.78
GC	56	765.97^{p}(578.6-994.67)	22	350.97^{p}(219.95-531.38)	32	217.20^{p}(148.56-306.62)	14	45.73^{p}(25-76.73)	124	210.43^{p}(175.02-250.89)	345	75.72
All Causes of Death	73	6.39^{p}(5.01-8.03)	32	3.21^{p}(2.19-4.53)	55	2.31^{p}(1.74-3)	85	1.43^{p}(1.14-1.77)	245	2.34^{p}(2.06-2.65)	345	78.85
Non-cancer causes	14	1.57(0.86-2.63)	8	1.02(0.44-2.01)	20	1.07(0.65-1.65)	58	1.22(0.93-1.58)	100	1.2(0.98-1.46)	345	82.55
Septicemia	0	0(0-21.72)	0	0(0-24.59)	0	0(0-10.07)	2	2.32(0.28-8.39)	2	1.29(0.16-4.67)	345	69.12
Other Infectious and Parasitic Diseases including HIV	0	0(0-41.28)	0	0(0-44.77)	1	4.86(0.12-27.06)	1	2.15(0.05-11.98)	2	2.37(0.29-8.57)	345	72.12
Diabetes Mellitus	0	0(0-9.51)	0	0(0-11.08)	1	1.28(0.03-7.14)	6	3.29^{p}(1.21-7.17)	7	2.11(0.85-4.34)	345	77.29
Alzheimer's (ICD-9 and 10 only)	0	0(0-9.74)	0	0(0-10.43)	0	0(0-4.03)	6	2.02(0.74-4.39)	6	1.3(0.48-2.83)	345	88.36
Cardiovascular Diseases	4	1.06(0.29-2.7)	3	0.92(0.19-2.7)	9	1.21(0.55-2.3)	21	1.2(0.75-1.84)	37	1.16(0.82-1.6)	345	84.36
Cerebrovascular Diseases	0	0(0-4.34)	1	1.41(0.04-7.88)	1	0.62(0.02-3.45)	1	0.26(0.01-1.47)	3	0.43(0.09-1.26)	345	83.03
Pneumonia and Influenza	0	0(0-9.92)	0	0(0-11.42)	1	1.44(0.04-8.02)	1	0.62(0.02-3.47)	2	0.67(0.08-2.41)	345	87.63
Chronic Obstructive Pulmonary Disease and Allied Cond	4	6.38^{p}(1.74-16.33)	2	3.67(0.44-13.27)	4	3(0.82-7.68)	2	0.6(0.07-2.16)	12	2.05^{p}(1.06-3.58)	345	81.26

Chronic Liver Disease and Cirrhosis	0	0(0-50.53)	0	0(0-60.79)	2	13.55^{p}(1.64-48.94)	0	0(0-10.33)	2	3.13(0.38-11.31)	345	69.34
Nephritis, Nephrotic Syndrome and Nephrosis	2	8.16(0.99-29.49)	0	0(0-16.51)	1	1.75(0.04-9.76)	2	1.48(0.18-5.34)	5	2.09(0.68-4.88)	345	78.82
Accidents and Adverse Effects	0	0(0-14.41)	0	0(0-16.06)	0	0(0-6.6)	3	1.9(0.39-5.56)	3	1.14(0.24-3.34)	345	81.39
Suicide and Self-Inflicted Injury	1	21.59(0.55-120.31)	0	0(0-97.34)	0	0(0-39.19)	0	0(0-16.16)	1	2.46(0.06-13.7)	345	65
Other Cause of Death	3	1.81(0.37-5.28)	2	1.3(0.16-4.68)	0	0.00^{p}(0-0.93)	13	1.1(0.59-1.89)	18	0.95(0.56-1.5)	345	85.47

Table S28: Standardized-mortality ratios (SMRs) for non-cancer causes for non-cardia during 2000-2004.

Causes	<1 years		1-2 years		2-5 years		>5 years		Total		Total	
	Observed (n)	SMR (95%CI)	Observed (n)	SMR (95%CI)	Observed (n)	Observed (n)	SMR (95%CI)	Observed (n)	SMR (95%CI)	Observed (n)	Patient	Mean Age at Event
Non-GC	24	1.91^{p}(1.22-2.83)	24	2.35^{p}(1.51-3.5)	22	0.87(0.55-1.32)	50	1.12(0.83-1.48)	120	1.30^{p}(1.08-1.55)	1815	79.65
GC	266	711.96^{p}(628.96-802.86)	98	323.99^{p}(263.03-394.83)	137	186.18^{p}(156.31-220.1)	53	43.60^{p}(32.66-57.03)	554	210.85^{p}(193.65-229.16)	1815	76.65
All Causes of Death	411	6.60^{p}(5.98-7.27)	165	3.26^{p}(2.79-3.8)	260	2.01^{p}(1.78-2.27)	360	1.43^{p}(1.29-1.59)	1196	2.42^{p}(2.29-2.56)	1815	79.88
Non-cancer causes	121	2.45^{p}(2.04-2.93)	43	1.07(0.78-1.45)	101	0.98(0.8-1.19)	257	1.25^{p}(1.1-1.41)	522	1.31^{p}(1.2-1.43)	1815	83.36
Septicemia	5	5.34^{p}(1.73-12.47)	0	0(0-4.93)	2	1.07(0.13-3.85)	4	1.12(0.31-2.88)	11	1.55(0.77-2.77)	1815	80.5
Other Infectious and Parasitic Diseases including HIV	4	7.62^{p}(2.08-19.5)	1	2.29(0.06-12.76)	3	2.73(0.56-7.97)	2	1.1(0.13-3.98)	10	2.58^{p}(1.24-4.74)	1815	74.25
Diabetes Mellitus	6	3.06^{p}(1.12-6.67)	0	0(0-2.34)	2	0.5(0.06-1.82)	6	0.8(0.3-1.75)	14	0.94(0.51-1.57)	1815	81.94
Alzheimer's (ICD-9 and 10 only)	4	1.56(0.43-4)	2	0.93(0.11-3.36)	1	0.17^{p}(0-0.96)	13	0.88(0.47-1.51)	20	0.79(0.48-1.22)	1815	92.68
Cardiovascular Diseases	46	2.35^{p}(1.72-3.14)	17	1.1(0.64-1.76)	28	0.73(0.48-1.05)	107	1.45^{p}(1.19-1.75)	198	1.34^{p}(1.16-1.54)	1815	84.39
Cerebrovascular Diseases	13	3.04^{p}(1.62-5.2)	3	0.88(0.18-2.58)	10	1.19(0.57-2.19)	22	1.32(0.82-1.99)	48	1.46^{p}(1.08-1.94)	1815	84.22
Pneumonia and Influenza	6	3.19^{p}(1.17-6.95)	3	2.03(0.42-5.93)	5	1.32(0.43-3.08)	5	0.7(0.23-1.63)	19	1.33(0.8-2.08)	1815	84.17
Chronic Obstructive Pulmonary Disease and Allied Cond	6	1.71(0.63-3.73)	1	0.35(0.01-1.95)	10	1.36(0.65-2.51)	11	0.8(0.4-1.43)	28	1.02(0.68-1.47)	1815	82.12

Chronic Liver Disease and Cirrhosis	3	8.24^{p}(1.7-24.08)	1	3.31(0.08-18.45)	4	5.26^{p}(1.43-13.47)	2	1.43(0.17-5.16)	10	3.54^{p}(1.7-6.51)	1815	67.53
Nephritis, Nephrotic Syndrome and Nephrosis	4	2.73(0.74-6.98)	1	0.84(0.02-4.68)	4	1.35(0.37-3.45)	7	1.26(0.51-2.6)	16	1.43(0.82-2.32)	1815	83.74
Accidents and Adverse Effects	1	0.68(0.02-3.8)	1	0.82(0.02-4.58)	4	1.24(0.34-3.18)	13	1.90^{p}(1.01-3.25)	19	1.49(0.9-2.33)	1815	76.38
Suicide and Self-Inflicted Injury	1	4.32(0.11-24.09)	0	0(0-19.05)	1	2.06(0.05-11.49)	0	0(0-4.19)	2	1.12(0.14-4.04)	1815	78.88
Other Cause of Death	22	2.07^{p}(1.3-3.14)	13	1.45(0.77-2.48)	27	1.09(0.72-1.58)	65	1.24(0.96-1.58)	127	1.31^{p}(1.09-1.56)	1815	83.56

Table S29: Standardized-mortality ratios (SMRs) for non-cancer causes for non-cardia during 2005-2009.

Causes	<1 years		1-2 years		2-5 years		>5 years		Total		Total	
	Observed (n)	SMR (95%CI)	Observed (n)	SMR (95%CI)	Observed (n)	Observed (n)	SMR (95%CI)	Observed (n)	SMR (95%CI)	Observed (n)	Patient	Mean Age at Event
Non-GC	19	1.69^{p}(1.02-2.64)	9	0.97(0.44-1.85)	25	1.1(0.71-1.62)	21	1.41(0.87-2.15)	74	1.27(1-1.6)	1848	74.88
GC	291	938.68^{p}(833.92-1052.96)	131	507.78^{p}(424.55-602.55)	121	195.21^{p}(161.98-233.25)	21	52.91^{p}(32.75-80.88)	564	355.90^{p}(327.13-386.52)	1848	76.84
All Causes of Death	389	7.08^{p}(6.4-7.82)	177	3.91^{p}(3.35-4.53)	248	2.15^{p}(1.89-2.44)	128	1.64^{p}(1.36-1.94)	942	3.21^{p}(3.01-3.42)	1848	77.68
Non-cancer causes	79	1.82^{p}(1.44-2.27)	37	1.03(0.73-1.43)	102	1.11(0.91-1.35)	86	1.37^{p}(1.09-1.69)	304	1.30^{p}(1.16-1.45)	1848	79.94
Septicemia	3	3.64(0.75-10.64)	1	1.47(0.04-8.2)	3	1.75(0.36-5.13)	5	4.56^{p}(1.48-10.65)	12	2.79^{p}(1.44-4.87)	1848	73.58
Other Infectious and Parasitic Diseases including HIV	2	4.1(0.5-14.8)	1	2.53(0.06-14.11)	4	4.30^{p}(1.17-11.02)	0	0(0-6.48)	7	2.94^{p}(1.18-6.05)	1848	76.19
Diabetes Mellitus	4	2.28(0.62-5.84)	4	2.75(0.75-7.04)	4	1.09(0.3-2.78)	1	0.39(0.01-2.18)	13	1.38(0.73-2.35)	1848	75
Alzheimer's (ICD-9 and 10 only)	0	0(0-1.56)	0	0(0-1.8)	3	0.5(0.1-1.47)	4	0.9(0.24-2.29)	7	0.47^{p}(0.19-0.97)	1848	88.19
Cardiovascular Diseases	32	2.03^{p}(1.39-2.86)	8	0.62(0.27-1.22)	33	1.01(0.7-1.42)	31	1.41(0.96-2)	104	1.25^{p}(1.02-1.51)	1848	81.98
Cerebrovascular Diseases	2	0.59(0.07-2.12)	2	0.71(0.09-2.56)	8	1.09(0.47-2.14)	8	1.54(0.66-3.03)	20	1.06(0.65-1.64)	1848	83.02
Pneumonia and Influenza	3	1.98(0.41-5.79)	1	0.8(0.02-4.47)	8	2.62^{p}(1.13-5.16)	7	3.57^{p}(1.44-7.35)	19	2.44^{p}(1.47-3.81)	1848	81.09
Chronic Obstructive Pulmonary Disease and Allied Cond	5	1.6(0.52-3.72)	4	1.54(0.42-3.95)	6	0.92(0.34-2)	5	1.17(0.38-2.72)	20	1.21(0.74-1.87)	1848	73.53

Chronic Liver Disease and Cirrhosis	2	5.18(0.63-18.7)	1	2.99(0.08-16.66)	5	5.89[p](1.91-13.73)	3	5.23[p](1.08-15.3)	11	5.13[p](2.56-9.18)	1848	67.38
Nephritis, Nephrotic Syndrome and Nephrosis	1	0.8(0.02-4.48)	4	3.98[p](1.09-10.2)	4	1.57(0.43-4.02)	5	2.9(0.94-6.76)	14	2.15[p](1.17-3.6)	1848	83.78
Accidents and Adverse Effects	1	0.69(0.02-3.82)	3	2.42(0.5-7.08)	4	1.22(0.33-3.12)	3	1.29(0.27-3.76)	11	1.32(0.66-2.37)	1848	78.75
Suicide and Self-Inflicted Injury	0	0(0-15.5)	0	0(0-18.24)	0	0(0-7.28)	0	0(0-10.88)	0	0(0-2.87)	1848	
Other Cause of Death	24	2.23[p](1.43-3.32)	8	0.9(0.39-1.77)	20	0.88(0.54-1.36)	14	0.89(0.48-1.48)	66	1.13(0.88-1.44)	1848	80.53

Table S30: Standardized-mortality ratios (SMRs) for non-cancer causes for non-cardia during 2010-2014.

Causes	<1 years		1-2 years		2-5 years		>5 years		Total		Total	
	Observed (n)	SMR (95%CI)	Observed (n)	SMR (95%CI)	Observed (n)	Observed (n)	SMR (95%CI)	Observed (n)	SMR (95%CI)	Observed (n)	Patient	Mean Age at Event
Non-GC	14	2.06[p](1.13-3.45)	5	1.2(0.39-2.8)	8	1.65(0.71-3.26)	0	0(0-0)	27	1.71[p](1.13-2.48)	1329	79.08
GC	167	970.17[p](828.61-1128.99)	50	470.22[p](349-619.92)	31	252.31[p](171.43-358.13)	0	0(0-0)	248	617.94[p](543.42-699.82)	1329	76.65
All Causes of Death	229	6.57[p](5.74-7.47)	73	3.42[p](2.68-4.29)	66	2.57[p](1.99-3.27)	0	0(0-0)	368	4.49[p](4.04-4.97)	1329	77.71
Non-cancer causes	48	1.72[p](1.27-2.28)	18	1.05(0.62-1.66)	27	1.3(0.86-1.9)	0	0(0-0)	93	1.42[p](1.14-1.73)	1329	80.13
Septicemia	3	5.87[p](1.21-17.17)	0	0(0-12.04)	0	0(0-10.4)	0	0(0-0)	3	2.56(0.53-7.48)	1329	82.38
Other Infectious and Parasitic Diseases including HIV	0	0(0-14.03)	0	0(0-23.22)	1	5.58(0.14-31.1)	0	0(0-0)	1	1.66(0.04-9.27)	1329	83.83
Diabetes Mellitus	2	1.79(0.22-6.46)	2	2.89(0.35-10.46)	2	2.44(0.3-8.8)	0	0(0-0)	6	2.28(0.84-4.97)	1329	74.52
Alzheimer's (ICD-9 and 10 only)	1	0.52(0.01-2.89)	0	0(0-3.11)	0	0(0-2.42)	0	0(0-0)	1	0.22(0.01-1.2)	1329	72
Cardiovascular Diseases	19	1.93[p](1.16-3.02)	7	1.17(0.47-2.41)	10	1.39(0.67-2.56)	0	0(0-0)	36	1.57[p](1.1-2.17)	1329	82.91
Cerebrovascular Diseases	4	1.79(0.49-4.58)	0	0(0-2.68)	5	2.92(0.95-6.82)	0	0(0-0)	9	1.69(0.77-3.21)	1329	77.4
Pneumonia and Influenza	1	1.17(0.03-6.54)	1	1.93(0.05-10.77)	1	1.69(0.04-9.4)	0	0(0-0)	3	1.53(0.32-4.47)	1329	89.36
Chronic Obstructive Pulmonary Disease and Allied Cond	5	2.57(0.83-6)	1	0.83(0.02-4.65)	2	1.42(0.17-5.15)	0	0(0-0)	8	1.76(0.76-3.46)	1329	79.41

Chronic Liver Disease and Cirrhosis	1	3.59(0.09-20)	0	0(0-21)	0	0(0-18.86)	0	0(0-0)	1	1.54(0.04-8.57)	1329	82.5
Nephritis, Nephrotic Syndrome and Nephrosis	2	2.59(0.31-9.35)	0	0(0-7.91)	0	0(0-6.63)	0	0(0-0)	2	1.11(0.13-4.02)	1329	76.92
Accidents and Adverse Effects	3	2.85(0.59-8.34)	0	0(0-5.64)	2	2.57(0.31-9.3)	0	0(0-0)	5	2.01(0.65-4.7)	1329	81.18
Suicide and Self-Inflicted Injury	0	0(0-21.91)	0	0(0-34.53)	0	0(0-32.28)	0	0(0-0)	0	0(0-9.47)	1329	
Other Cause of Death	7	1.01(0.41-2.08)	7	1.63(0.66-3.37)	4	0.75(0.21-1.93)	0	0(0-0)	18	1.09(0.64-1.72)	1329	76.39

Table S31: Standardized-mortality ratios (SMRs) for non-cancer causes for non-cardia during 2015-2019.

Causes	<1 years		1-2 years		2-5 years		>5 years		Total		Total	
	Observed (n)	SMR (95%CI)	Observed (n)	SMR (95%CI)	Observed (n)	Observed (n)	SMR (95%CI)	Observed (n)	SMR (95%CI)	Observed (n)	Patient	Mean Age at Event
Non-GC	24	1.35(0.87-2.01)	19	1.33(0.8-2.08)	35	1.05(0.73-1.46)	52	1.14(0.85-1.49)	130	1.17(0.98-1.39)	2885	77.91
GC	336	647.21[P](579.85-720.24)	156	371.79[P](315.74-434.93)	163	168.88[P](143.95-196.89)	50	39.64[P](29.42-52.26)	705	222.72[P](206.58-239.79)	2885	74.15
All Causes of Death	460	5.88[P](5.36-6.45)	230	3.69[P](3.23-4.2)	313	2.07[P](1.85-2.32)	313	1.34[P](1.19-1.49)	1316	2.50[P](2.37-2.64)	2885	77.27
Non-cancer causes	100	1.67[P](1.36-2.03)	55	1.15(0.87-1.5)	115	0.99(0.81-1.18)	211	1.13(0.98-1.29)	481	1.17[P](1.07-1.28)	2885	81.67
Septicemia	5	4.39[P](1.43-10.25)	0	0(0-4.05)	2	0.91(0.11-3.29)	7	2.12(0.85-4.36)	14	1.86[P](1.01-3.11)	2885	82.28
Other Infectious and Parasitic Diseases including HIV	1	1.43(0.04-7.96)	1	1.78(0.05-9.92)	3	2.28(0.47-6.66)	3	1.69(0.35-4.95)	8	1.84(0.79-3.62)	2885	79.91
Diabetes Mellitus	2	0.77(0.09-2.78)	5	2.39(0.78-5.58)	6	1.2(0.44-2.62)	5	0.68(0.22-1.59)	18	1.06(0.63-1.67)	2885	77.27
Alzheimer's (ICD-9 and 10 only)	1	0.37(0.01-2.05)	1	0.46(0.01-2.55)	1	0.17[P](0-0.95)	10	0.85(0.41-1.57)	13	0.58[P](0.31-0.99)	2885	88.25
Cardiovascular Diseases	43	1.88[P](1.36-2.54)	19	1.06(0.64-1.66)	38	0.88(0.63-1.21)	79	1.17(0.92-1.45)	179	1.18[P](1.01-1.37)	2885	82.62
Cerebrovascular Diseases	7	1.48(0.59-3.04)	2	0.53(0.06-1.93)	9	0.99(0.45-1.88)	18	1.22(0.73-1.94)	36	1.12(0.78-1.54)	2885	81.89
Pneumonia and Influenza	1	0.46(0.01-2.58)	4	2.36(0.64-6.05)	10	2.43[P](1.17-4.48)	8	1.24(0.53-2.44)	23	1.59[P](1.01-2.39)	2885	83.72
Chronic Obstructive Pulmonary Disease and Allied Cond	7	1.5(0.6-3.1)	4	1.07(0.29-2.74)	12	1.33(0.69-2.33)	8	0.6(0.26-1.18)	31	1.01(0.69-1.43)	2885	79.59

Chronic Liver Disease and Cirrhosis	4	6.10[P](1.66-15.63)	0	0(0-6.96)	4	3.28(0.89-8.4)	3	1.91(0.39-5.57)	11	2.76[P](1.38-4.95)	2885	71.52
Nephritis, Nephrotic Syndrome and Nephrosis	3	1.7(0.35-4.98)	4	2.85(0.78-7.29)	3	0.88(0.18-2.57)	8	1.53(0.66-3.01)	18	1.52(0.9-2.41)	2885	83.2
Accidents and Adverse Effects	2	0.92(0.11-3.32)	4	2.29(0.62-5.85)	2	0.47(0.06-1.7)	10	1.49(0.72-2.74)	18	1.21(0.72-1.91)	2885	76.86
Suicide and Self-Inflicted Injury	2	4.49(0.54-16.23)	0	0(0-10.23)	1	1.22(0.03-6.79)	0	0(0-3.45)	3	1.11(0.23-3.25)	2885	74.25
Other Cause of Death	22	1.64[P](1.03-2.49)	11	1.02(0.51-1.83)	24	0.88(0.56-1.31)	52	1.12(0.84-1.47)	109	1.11(0.92-1.34)	2885	81.94

Table S32: Standardized-mortality ratios (SMRs) for non-cancer causes for non-cardia in married patients.

Causes	<1 years		1-2 years		2-5 years		>5 years		Total		Total	
	Observed (n)	SMR (95%CI)	Observed (n)	SMR (95%CI)	Observed (n)	Observed (n)	SMR (95%CI)	Observed (n)	SMR (95%CI)	Observed (n)	Patient	Mean Age at Event
Non-GC	31	2.26[P](1.53-3.21)	18	1.76[P](1.05-2.79)	19	0.87(0.53-1.36)	28	1.26(0.84-1.82)	96	1.41[P](1.14-1.73)	2198	78.82
GC	406	1,100.93[P](996.43-1213.41)	134	482.73[P](404.46-571.73)	142	241.66[P](203.55-284.84)	34	58.17[P](40.28-81.29)	716	393.74[P](365.42-423.67)	2198	79.08
All Causes of Death	588	7.62[P](7.02-8.26)	200	3.44[P](2.98-3.95)	279	2.19[P](1.94-2.47)	237	1.72[P](1.51-1.95)	1304	3.26[P](3.08-3.44)	2198	80.24
Non-cancer causes	151	2.39[P](2.03-2.81)	48	1.01(0.74-1.33)	118	1.13(0.93-1.35)	175	1.52[P](1.3-1.76)	492	1.49[P](1.36-1.63)	2198	82.21
Septicemia	6	5.08[P](1.87-11.06)	1	1.14(0.03-6.36)	3	1.59(0.33-4.64)	4	2.03(0.55-5.19)	14	2.37[P](1.29-3.97)	2198	71.57
Other Infectious and Parasitic Diseases including HIV	5	8.31[P](2.7-19.4)	1	2.18(0.06-12.13)	6	6.10[P](2.24-13.29)	0	0(0-3.82)	12	3.99[P](2.06-6.97)	2198	72.06
Diabetes Mellitus	10	4.22[P](2.02-7.76)	1	0.57(0.01-3.15)	3	0.79(0.16-2.3)	8	1.99(0.86-3.93)	22	1.84[P](1.15-2.79)	2198	78.16
Alzheimer's (ICD-9 and 10 only)	4	0.97(0.26-2.47)	1	0.31(0.01-1.73)	3	0.4(0.08-1.17)	13	1.38(0.74-2.37)	21	0.87(0.54-1.33)	2198	91.7
Cardiovascular Diseases	55	2.32[P](1.75-3.02)	16	0.9(0.52-1.47)	34	0.89(0.62-1.25)	71	1.75[P](1.37-2.21)	176	1.47[P](1.26-1.7)	2198	84.4
Cerebrovascular Diseases	12	2.18[P](1.13-3.81)	4	0.97(0.26-2.48)	11	1.23(0.61-2.2)	11	1.11(0.56-1.99)	38	1.33(0.94-1.83)	2198	85.33
Pneumonia and Influenza	8	3.58[P](1.55-7.06)	1	0.59(0.01-3.3)	4	1.12(0.3-2.86)	5	1.33(0.43-3.09)	18	1.6(0.95-2.52)	2198	82.91
Chronic Obstructive Pulmonary Disease and Allied Cond	11	2.70[P](1.35-4.82)	4	1.3(0.35-3.33)	10	1.49(0.72-2.75)	10	1.41(0.67-2.59)	35	1.67[P](1.16-2.32)	2198	78.56

Chronic Liver Disease and Cirrhosis	2	5.06(0.61-18.27)	2	6.63(0.8-23.96)	7	10.78^{p}(4.34-22.22)	2	2.97(0.36-10.74)	13	6.44^{p}(3.43-11.01)	2198	65.45
Nephritis, Nephrotic Syndrome and Nephrosis	6	3.37^{p}(1.24-7.33)	1	0.75(0.02-4.19)	6	2.08(0.76-4.52)	6	1.99(0.73-4.33)	19	2.11^{p}(1.27-3.29)	2198	82.27
Accidents and Adverse Effects	2	1.08(0.13-3.92)	0	0(0-2.62)	7	2.22(0.89-4.58)	7	1.96(0.79-4.05)	16	1.6(0.92-2.61)	2198	79.67
Suicide and Self-Inflicted Injury	0	0(0-17.94)	0	0(0-23.95)	0	0(0-11.43)	0	0(0-11.46)	0	0(0-3.67)	2198	
Other Cause of Death	30	1.99^{p}(1.35-2.85)	16	1.38(0.79-2.24)	24	0.91(0.58-1.36)	38	1.27(0.9-1.74)	108	1.30^{p}(1.07-1.57)	2198	82.46

Table S33: Standardized-mortality ratios (SMRs) for non-cancer causes for non-cardia in unmarried patients.

Causes	<1 years		1-2 years		2-5 years		>5 years		Total		Total	
	Observed (n)	SMR (95%CI)	Observed (n)	SMR (95%CI)	Observed (n)	Observed (n)	SMR (95%CI)	Observed (n)	SMR (95%CI)	Observed (n)	Patient	Mean Age at Event
Non-GC	5	3.11^{p}(1.01-7.27)	3	2.35(0.49-6.88)	4	1.39(0.38-3.56)	4	1.4(0.38-3.59)	16	1.86^{p}(1.06-3.02)	254	75.88
GC	38	928.17^{p}(656.83-1273.98)	11	340.49^{p}(169.97-609.22)	16	218.86^{p}(125.1-355.42)	4	55.05^{p}(15-140.94)	69	315.04^{p}(245.12-398.7)	254	76.9
All Causes of Death	54	6.63^{p}(4.98-8.65)	17	2.54^{p}(1.48-4.06)	37	2.33^{p}(1.64-3.22)	23	1.35(0.85-2.02)	131	2.74^{p}(2.29-3.25)	254	78.75
Non-cancer causes	11	1.69(0.84-3.03)	3	0.56(0.11-1.63)	17	1.32(0.77-2.11)	15	1.06(0.59-1.75)	46	1.18(0.86-1.57)	254	82.53
Septicemia	0	0(0-30.25)	0	0(0-37.84)	0	0(0-16.41)	0	0(0-15.79)	0	0(0-5.44)	254	
Other Infectious and Parasitic Diseases including HIV	0	0(0-57.48)	0	0(0-71.33)	0	0(0-31.99)	0	0(0-31.91)	0	0(0-10.64)	254	
Diabetes Mellitus	0	0(0-14.88)	0	0(0-18.7)	0	0(0-8.22)	0	0(0-7.86)	0	0(0-2.71)	254	
Alzheimer's (ICD-9 and 10 only)	0	0(0-9.74)	0	0(0-10.9)	0	0(0-4.18)	0	0(0-3.46)	0	0(0-1.38)	254	
Cardiovascular Diseases	3	1.24(0.25-3.61)	0	0(0-1.85)	8	1.69(0.73-3.33)	9	1.77(0.81-3.37)	20	1.41(0.86-2.17)	254	84.84
Cerebrovascular Diseases	0	0(0-7.08)	0	0(0-8.53)	4	3.89^{p}(1.06-9.95)	2	1.75(0.21-6.33)	6	1.92(0.7-4.18)	254	76.35
Pneumonia and Influenza	1	4.47(0.11-24.93)	0	0(0-19.98)	1	2.29(0.06-12.73)	0	0(0-7.82)	2	1.52(0.18-5.48)	254	82.63

Chronic Obstructive Pulmonary Disease and Allied Cond	2	4.24(0.51-15.32)	0	0(0-9.61)	0	0(0-4.07)	0	0(0-3.93)	2	0.74(0.09-2.68)	254	81.8
Chronic Liver Disease and Cirrhosis	0	0(0-72.22)	0	0(0-89.49)	0	0(0-43.47)	0	0(0-43.86)	0	0(0-14.12)	254	
Nephritis, Nephrotic Syndrome and Nephrosis	0	0(0-19.77)	0	0(0-24.55)	0	0(0-10.45)	0	0(0-9.95)	0	0(0-3.48)	254	
Accidents and Adverse Effects	1	4.74(0.12-26.44)	0	0(0-20.69)	1	2.32(0.06-12.93)	2	4.08(0.49-14.74)	4	3.05(0.83-7.82)	254	77.3
Suicide and Self-Inflicted Injury	0	0(0-111.54)	0	0(0-141.7)	0	0(0-64.97)	0	0(0-64.09)	0	0(0-21.27)	254	
Other Cause of Death	4	2.56(0.7-6.54)	3	2.29(0.47-6.7)	3	0.93(0.19-2.73)	2	0.55(0.07-1.98)	12	1.23(0.64-2.15)	254	83.62

Table S34: Standardized-mortality ratios (SMRs) for non-cancer causes for non-cardia in unknown marital status patients.

causes	<1 years		1-2 years		2-5 years		>5 years		Total	
	Observed (n)	SMR (95%CI)	Observed (n)	SMR (95%CI)	Observed (n)	Observed (n)	SMR (95%CI)	Observed (n)	SMR (95%CI)	Observed (n)
All Causes of Death	1063	9.00[p](8.46-9.55)	550	6.70[p](6.15-7.28)	660	3.92[p](3.62-4.23)	357	1.85[p](1.67-2.06)	2630	4.68[p](4.51-4.87)
All Malignant Cancers	850	30.19[p](28.2-32.29)	443	22.15[p](20.13-24.31)	464	11.53[p](10.51-12.63)	159	3.71[p](3.16-4.33)	1916	14.60[p](13.95-15.27)
Oral Cavity and Pharynx	1	2.21(0.06-12.32)	0	0(0-11.2)	0	0(0-5.46)	0	0(0-5.07)	1	0.46(0.01-2.55)
Digestive System	828	114.84[p](107.15-122.94)	428	82.85[p](75.19-91.08)	426	40.84[p](37.06-44.91)	126	11.33[p](9.44-13.49)	1808	53.29[p](50.86-55.81)
Esophagus	471	527.82[p](481.22-577.71)	264	408.07[p](360.32-460.38)	281	215.15[p](190.73-241.84)	78	56.91[p](44.98-71.02)	1094	259.48[p](244.33-275.33)
Stomach	347	609.40[p](546.96-677.01)	154	383.57[p](325.39-449.17)	128	163.53[p](136.43-194.44)	36	45.34[p](31.75-62.76)	665	261.02[p](241.56-281.64)
Small Intestine	0	0(0-58.96)	0	0(0-80.75)	0	0(0-38.81)	0	0(0-33.71)	0	0(0-11.8)
Colon and Rectum	2	0.81(0.1-2.94)	3	1.75(0.36-5.11)	7	2.06(0.83-4.25)	3	0.85(0.18-2.49)	15	1.35(0.76-2.23)

Colon excluding Rectum	2	1(0.12-3.6)	3	2.15(0.44-6.28)	6	2.18(0.8-4.75)	2	0.7(0.09-2.54)	13	1.45(0.77-2.47)
Rectum and Rectosigmoid Junction	0	0(0-8.19)	0	0(0-11.49)	1	1.54(0.04-8.61)	1	1.47(0.04-8.19)	2	0.95(0.12-3.44)
Anus, Anal Canal and Anorectum	0	0(0-132.42)	0	0(0-178.78)	0	0(0-83.99)	0	0(0-71.89)	0	0(0-25.67)
Liver and Intrahepatic Bile Duct	0	0(0-3.31)	3	3.63(0.75-10.62)	3	1.76(0.36-5.13)	1	0.54(0.01-2.99)	7	1.27(0.51-2.62)
Liver	0	0(0-4.25)	3	4.67(0.96-13.64)	3	2.27(0.47-6.64)	0	0(0-2.6)	6	1.41(0.52-3.07)
Intrahepatic Bile Duct	0	0(0-14.95)	0	0(0-20.17)	0	0(0-9.52)	1	2.23(0.06-12.45)	1	0.79(0.02-4.41)
Gallbladder	1	12.44(0.32-69.34)	0	0(0-64.72)	0	0(0-32.09)	0	0(0-30.39)	1	2.68(0.07-14.91)
Other Biliary	0	0(0-46.38)	0	0(0-65.67)	0	0(0-31.7)	1	7.52(0.19-41.89)	1	2.6(0.07-14.47)
Pancreas	3	1.64(0.34-4.79)	3	2.26(0.47-6.6)	4	1.47(0.4-3.76)	5	1.67(0.54-3.91)	15	1.69(0.95-2.79)
Retroperitoneum	0	0(0-334.58)	0	0(0-466.94)	0	0(0-222.89)	0	0(0-193.82)	0	0(0-67.68)
Peritoneum, Omentum and Mesentery	0	0(0-152.27)	0	0(0-207.01)	0	0(0-98.95)	0	0(0-86.43)	0	0(0-30.23)
Other Digestive Organs	4	69.39^{p}(18.91-177.66)	1	23.59(0.6-131.44)	3	33.79^{p}(6.97-98.75)	2	19.21^{p}(2.33-69.4)	10	34.14^{p}(16.37-62.78)
Respiratory System	2	0.24^{p}(0.03-0.87)	5	0.85(0.28-1.98)	14	1.21(0.66-2.03)	13	1.12(0.6-1.91)	34	0.91(0.63-1.27)
Nose, Nasal Cavity and Middle Ear	0	0(0-166.41)	0	0(0-233.06)	0	0(0-113.52)	0	0(0-108.8)	0	0(0-35.33)
Larynx	0	0(0-16.95)	0	0(0-23.65)	0	0(0-11.96)	0	0(0-11.87)	0	0(0-3.72)
Lung and Bronchus	2	0.25^{p}(0.03-0.9)	5	0.88(0.28-2.05)	14	1.25(0.68-2.1)	13	1.16(0.62-1.98)	34	0.94(0.65-1.31)
Pleura	0	0(0-203.17)	0	0(0-283.9)	0	0(0-134.47)	0	0(0-106.64)	0	0(0-39.59)
Trachea, Mediastinum and Other Respiratory Organs	0	0(0-344.51)	0	0(0-481.31)	0	0(0-232.58)	0	0(0-206.02)	0	0(0-70.75)
Bones and Joints	0	0(0-76.27)	0	0(0-105.55)	0	0(0-50.58)	0	0(0-43.27)	0	0(0-15.27)
Soft Tissue including Heart	0	0(0-21.53)	0	0(0-29.74)	1	3.95(0.1-22.01)	0	0(0-13.38)	1	1.21(0.03-6.76)

Skin excluding Basal and Squamous	0	0(0-5.43)	0	0(0-7.58)	3	3.01(0.62-8.79)	3	2.7(0.56-7.9)	6	1.83(0.67-3.99)
Melanoma of the Skin	0	0(0-7.85)	0	0(0-10.98)	3	4.43(0.91-12.94)	3	4.17(0.86-12.18)	6	2.72(1-5.93)
Other Non-Epithelial Skin	0	0(0-17.55)	0	0(0-24.5)	0	0(0-11.53)	0	0(0-9.46)	0	0(0-3.45)
Breast	0	0(0-5.59)	0	0(0-7.96)	0	0(0-3.94)	0	0(0-3.73)	0	0(0-1.21)
Female Genital System	1	2.16(0.05-12.06)	0	0(0-11.18)	0	0(0-5.52)	0	0(0-5.29)	1	0.46(0.01-2.58)
Cervix Uteri	0	0(0-90.17)	0	0(0-126.21)	0	0(0-63.55)	0	0(0-66.59)	0	0(0-20.09)
Corpus and Uterus, NOS	0	0(0-25.52)	0	0(0-35)	0	0(0-16.84)	0	0(0-15.68)	0	0(0-5.24)
Corpus Uteri	0	0(0-55.42)	0	0(0-75.37)	0	0(0-35.3)	0	0(0-30.87)	0	0(0-10.87)
Uterus, NOS	0	0(0-47.29)	0	0(0-65.36)	0	0(0-32.2)	0	0(0-31.88)	0	0(0-10.11)
Ovary	1	4.17(0.11-23.24)	0	0(0-21.78)	0	0(0-11)	0	0(0-10.85)	1	0.92(0.02-5.14)
Vagina	0	0(0-474.65)	0	0(0-688.55)	0	0(0-346.03)	0	0(0-318.62)	0	0(0-104.3)
Vulva	0	0(0-185.63)	0	0(0-268.84)	0	0(0-126.2)	0	0(0-102.78)	0	0(0-37.37)
Other Female Genital Organs	0	0(0-408.14)	0	0(0-537.65)	0	0(0-239.95)	0	0(0-196.67)	0	0(0-73.73)
Male Genital System	0	0(0-1.47)	0	0(0-2.13)	3	0.85(0.17-2.48)	3	0.75(0.15-2.18)	6	0.51(0.19-1.11)
Prostate	0	0(0-1.48)	0	0(0-2.16)	2	0.57(0.07-2.07)	3	0.76(0.16-2.21)	5	0.43(0.14-1)
Testis	0	0(0-460.13)	0	0(0-625.38)	1	85.76[p](2.17-477.82)	0	0(0-316.75)	1	26.87(0.68-149.69)
Penis	0	0(0-189.46)	0	0(0-260.96)	0	0(0-125.37)	0	0(0-108.61)	0	0(0-38.03)
Other Male Genital Organs	0	0(0-909.99)	0	0(0-1254.63)	0	0(0-591.86)	0	0(0-500.17)	0	0(0-179.05)
Urinary System	2	1.1(0.13-3.99)	1	0.78(0.02-4.34)	4	1.51(0.41-3.88)	4	1.34(0.37-3.44)	11	1.26(0.63-2.26)
Urinary Bladder	1	0.97(0.02-5.39)	0	0(0-5.07)	1	0.66(0.02-3.69)	2	1.13(0.14-4.1)	4	0.79(0.22-2.03)

Kidney and Renal Pelvis	1	1.37(0.03-7.65)	1	1.91(0.05-10.66)	3	2.83(0.58-8.28)	2	1.77(0.21-6.38)	7	2.03(0.82-4.19)
Ureter	0	0(0-160.44)	0	0(0-226.47)	0	0(0-109.55)	0	0(0-93.5)	0	0(0-32.82)
Other Urinary Organs	0	0(0-144.45)	0	0(0-200.08)	0	0(0-96.05)	0	0(0-82.82)	0	0(0-29.06)
Eye and Orbit	0	0(0-275.85)	0	0(0-380.78)	0	0(0-183.91)	0	0(0-162.04)	0	0(0-55.99)
Brain and Other Nervous System	0	0(0-6.19)	0	0(0-8.41)	1	1.12(0.03-6.21)	1	1.05(0.03-5.85)	2	0.69(0.08-2.51)
Endocrine System	1	9.27(0.23-51.68)	0	0(0-47.09)	0	0(0-23.03)	1	5.63(0.14-31.37)	2	3.82(0.46-13.79)
Thyroid	0	0(0-46.51)	0	0(0-64.05)	0	0(0-31.34)	1	7.56(0.19-42.13)	1	2.58(0.07-14.4)
Other Endocrine including Thymus	1	35.08(0.89-195.44)	0	0(0-177.89)	0	0(0-86.89)	0	0(0-81.36)	1	7.3(0.18-40.66)
Lymphoma	1	0.88(0.02-4.89)	0	0(0-4.6)	0	0(0-2.27)	2	1.12(0.14-4.04)	3	0.56(0.12-1.64)
Hodgkin Lymphoma	0	0(0-80.66)	0	0(0-114.16)	0	0(0-57.03)	0	0(0-54.38)	0	0(0-17.52)
Non-Hodgkin Lymphoma	1	0.91(0.02-5.09)	0	0(0-4.79)	0	0(0-2.37)	2	1.16(0.14-4.2)	3	0.58(0.12-1.71)
Myeloma	0	0(0-6.19)	0	0(0-8.68)	0	0(0-4.26)	1	1.05(0.03-5.83)	1	0.35(0.01-1.96)
Leukemia	0	0(0-3.03)	2	2.32(0.28-8.4)	2	1.14(0.14-4.12)	1	0.51(0.01-2.85)	5	0.86(0.28-2.02)
Lymphocytic Leukemia	0	0(0-10.92)	0	0(0-15.73)	0	0(0-7.74)	0	0(0-6.82)	0	0(0-2.32)
Acute Lymphocytic Leukemia	0	0(0-93.85)	0	0(0-129.16)	0	0(0-63.13)	0	0(0-58.25)	0	0(0-19.45)
Chronic Lymphocytic Leukemia	0	0(0-13.46)	0	0(0-19.52)	0	0(0-9.62)	0	0(0-8.48)	0	0(0-2.88)
Other Lymphocytic Leukemia	0	0(0-151.7)	0	0(0-218.03)	0	0(0-105.48)	0	0(0-87.22)	0	0(0-31.13)
Myeloid and Monocytic Leukemia	0	0(0-6.13)	1	2.3(0.06-12.83)	0	0(0-4.11)	1	0.98(0.02-5.46)	2	0.68(0.08-2.45)
Acute Myeloid Leukemia	0	0(0-7.47)	1	2.79(0.07-15.56)	0	0(0-4.99)	1	1.21(0.03-6.72)	2	0.83(0.1-2.98)
Acute Monocytic Leukemia	0	0(0-668.62)	0	0(0-946.84)	0	0(0-465.6)	0	0(0-414.82)	0	0(0-140.64)

Chronic Myeloid Leukemia	0	0(0-68.48)	0	0(0-98.12)	0	0(0-47.53)	0	0(0-40.59)	0	0(0-14.19)
Other Myeloid/Monocytic Leukemia	0	0(0-76.67)	0	0(0-106.25)	0	0(0-50.02)	0	0(0-40.85)	0	0(0-14.94)
Other Leukemia	0	0(0-13.27)	1	5.22(0.13-29.06)	2	5.29(0.64-19.1)	0	0(0-9.33)	3	2.41(0.5-7.05)
Other Acute Leukemia	0	0(0-36.87)	1	14.7(0.37-81.93)	2	15.32^{p}(1.86-55.35)	0	0(0-28.47)	3	7.01^{p}(1.44-20.48)
Aleukemic, Subleukemic and NOS	0	0(0-20.74)	0	0(0-29.81)	0	0(0-14.88)	0	0(0-13.88)	0	0(0-4.53)
Miscellaneous Malignant Cancer	14	6.42^{p}(3.51-10.77)	7	4.55^{p}(1.83-9.37)	10	3.21^{p}(1.54-5.91)	4	1.18(0.32-3.03)	35	3.43^{p}(2.39-4.76)

Table S35: Standardized-mortality ratios (SMRs) for cancer causes for cardia in all patients.

causes	<1 years		1-2 years		2-5 years		>5 years		Total	
	Observed (n)	SMR (95%CI)	Observed (n)	SMR (95%CI)	Observed (n)	Observed (n)	SMR (95%CI)	Observed (n)	SMR (95%CI)	Observed (n)
All Causes of Death	1102	6.74^{p}(6.35-7.15)	447	3.51^{p}(3.2-3.86)	629	2.14^{p}(1.98-2.31)	573	1.47^{p}(1.35-1.6)	2751	2.82^{p}(2.72-2.93)
All Malignant Cancers	840	24.72^{p}(23.07-26.45)	341	12.89^{p}(11.56-14.34)	379	6.36^{p}(5.74-7.04)	172	2.36^{p}(2.02-2.74)	1732	8.99^{p}(8.57-9.42)
Oral Cavity and Pharynx	0	0(0-7.81)	0	0(0-9.89)	1	1.18(0.03-6.57)	0	0(0-3.44)	1	0.36(0.01-2.01)
Tongue	0	0(0-34.31)	0	0(0-43.35)	1	5.13(0.13-28.57)	0	0(0-14.79)	1	1.57(0.04-8.75)
Digestive System	812	88.56^{p}(82.57-94.87)	322	44.70^{p}(39.95-49.86)	347	21.29^{p}(19.11-23.66)	113	5.66^{p}(4.66-6.8)	1594	30.28^{p}(28.81-31.8)
Esophagus	13	16.83^{p}(8.96-28.78)	8	13.28^{p}(5.73-26.17)	2	1.49(0.18-5.39)	1	0.61(0.02-3.42)	24	5.53^{p}(3.54-8.22)
Stomach	780	839.73^{p}(781.82-900.78)	301	412.62^{p}(367.32-461.97)	321	197.43^{p}(176.42-220.25)	88	45.87^{p}(36.79-56.51)	1490	286.38^{p}(272.02-301.3)
Small Intestine	1	12.9(0.33-71.86)	0	0(0-60.02)	0	0(0-25.58)	2	10.40^{p}(1.26-37.56)	3	6.31^{p}(1.3-18.44)

Colon and Rectum	5	1.54(0.5-3.58)	5	1.99(0.64-4.64)	3	0.54(0.11-1.57)	4	0.6(0.16-1.54)	17	0.94(0.55-1.51)
Colon excluding Rectum	5	1.84(0.6-4.3)	4	1.91(0.52-4.9)	3	0.65(0.13-1.89)	4	0.73(0.2-1.86)	16	1.07(0.61-1.74)
Rectum and Rectosigmoid Junction	0	0(0-6.81)	1	2.35(0.06-13.1)	0	0(0-3.86)	0	0(0-3.15)	1	0.32(0.01-1.8)
Liver and Intrahepatic Bile Duct	3	2.07(0.43-6.05)	2	1.71(0.21-6.18)	2	0.74(0.09-2.66)	3	0.87(0.18-2.55)	10	1.14(0.55-2.1)
Liver	3	2.68(0.55-7.83)	1	1.11(0.03-6.17)	1	0.48(0.01-2.68)	1	0.39(0.01-2.15)	6	0.9(0.33-1.95)
Intrahepatic Bile Duct	0	0(0-11.18)	1	3.75(0.09-20.9)	1	1.58(0.04-8.78)	2	2.36(0.29-8.54)	4	1.93(0.52-4.93)
Gallbladder	0	0(0-25.9)	0	0(0-32.96)	0	0(0-14.36)	1	3.28(0.08-18.26)	1	1.23(0.03-6.83)
Other Biliary	0	0(0-33.33)	0	0(0-42.16)	2	9.94[P](1.2-35.92)	0	0(0-14.06)	2	3.02(0.37-10.92)
Pancreas	5	2.2(0.72-5.14)	4	2.23(0.61-5.71)	12	2.91[P](1.51-5.09)	12	2.32[P](1.2-4.06)	33	2.47[P](1.7-3.47)
Other Digestive Organs	5	66.91[P](21.72-156.14)	2	32.81[P](3.97-118.52)	5	34.45[P](11.19-80.4)	2	10.15[P](1.23-36.66)	14	29.29[P](16.02-49.15)
Respiratory System	7	0.76(0.31-1.57)	4	0.56(0.15-1.43)	8	0.51[P](0.22-1)	21	1.16(0.72-1.77)	40	0.8(0.57-1.08)
Nose, Nasal Cavity and Middle Ear	0	0(0-139.15)	0	0(0-182.89)	0	0(0-81.62)	0	0(0-65.83)	0	0(0-24.94)
Larynx	0	0(0-17.51)	0	0(0-22.68)	1	2.82(0.07-15.73)	1	2.39(0.06-13.3)	2	1.74(0.21-6.3)
Lung and Bronchus	7	0.78(0.32-1.61)	4	0.58(0.16-1.47)	7	0.46[P](0.18-0.94)	20	1.14(0.69-1.76)	38	0.78(0.55-1.07)
Soft Tissue including Heart	0	0(0-18.79)	2	12.87[P](1.56-46.47)	1	2.84(0.07-15.81)	2	4.55(0.55-16.44)	5	4.37[P](1.42-10.2)
Skin excluding Basal and Squamous	0	0(0-6.43)	0	0(0-8.2)	0	0(0-3.57)	1	0.74(0.02-4.11)	1	0.29(0.01-1.63)
Melanoma of the Skin	0	0(0-9.73)	0	0(0-12.51)	0	0(0-5.53)	0	0(0-4.46)	0	0(0-1.7)
Other Non-Epithelial Skin	0	0(0-18.92)	0	0(0-23.85)	0	0(0-10.09)	1	1.89(0.05-10.51)	1	0.8(0.02-4.47)
Breast	2	1.22(0.15-4.4)	0	0(0-2.89)	0	0(0-1.28)	3	0.9(0.19-2.64)	5	0.55(0.18-1.28)
Female Genital System	0	0(0-3.07)	1	1.07(0.03-5.94)	1	0.47(0.01-2.64)	0	0(0-1.56)	2	0.3(0.04-1.09)

Cervix Uteri	0	0(0-30.5)	0	0(0-39.23)	0	0(0-17.81)	0	0(0-16.53)	0	0(0-5.72)
Corpus and Uterus, NOS	0	0(0-9.3)	0	0(0-11.89)	1	1.4(0.04-7.77)	0	0(0-4.36)	1	0.44(0.01-2.45)
Corpus Uteri	0	0(0-20.37)	0	0(0-26.25)	0	0(0-11.22)	0	0(0-9.04)	0	0(0-3.49)
Uterus, NOS	0	0(0-17.11)	0	0(0-21.74)	1	2.58(0.07-14.36)	0	0(0-8.4)	1	0.82(0.02-4.6)
Ovary	0	0(0-6.23)	1	2.17(0.05-12.07)	0	0(0-3.64)	0	0(0-3.43)	1	0.32(0.01-1.77)
Vagina	0	0(0-176.99)	0	0(0-224.47)	0	0(0-100.57)	0	0(0-85.94)	0	0(0-31.56)
Vulva	0	0(0-77.69)	0	0(0-97.53)	0	0(0-41.25)	0	0(0-32.6)	0	0(0-12.81)
Other Female Genital Organs	0	0(0-164.56)	0	0(0-202.77)	0	0(0-83.84)	0	0(0-65.96)	0	0(0-26.25)
Male Genital System	0	0(0-1.32)	0	0(0-1.76)	3	0.63(0.13-1.84)	2	0.31(0.04-1.13)	5	0.31[p](0.1-0.73)
Prostate	0	0(0-1.33)	0	0(0-1.78)	3	0.64(0.13-1.86)	2	0.32(0.04-1.14)	5	0.31[p](0.1-0.73)
Testis	0	0(0-653.65)	0	0(0-854.09)	0	0(0-378.07)	0	0(0-313.05)	0	0(0-117.09)
Penis	0	0(0-209.15)	0	0(0-272.57)	0	0(0-113.95)	0	0(0-80.41)	0	0(0-33.71)
Other Male Genital Organs	0	0(0-966.54)	0	0(0-1245.73)	0	0(0-542.98)	0	0(0-366.66)	0	0(0-156.09)
Urinary System	2	1.06(0.13-3.81)	0	0(0-2.5)	0	0(0-1.08)	4	0.89(0.24-2.28)	6	0.53(0.2-1.16)
Urinary Bladder	1	0.93(0.02-5.16)	0	0(0-4.41)	0	0(0-1.89)	2	0.75(0.09-2.7)	3	0.46(0.09-1.34)
Kidney and Renal Pelvis	1	1.32(0.03-7.34)	0	0(0-6.2)	0	0(0-2.74)	1	0.6(0.02-3.34)	2	0.46(0.06-1.65)
Ureter	0	0(0-133.16)	0	0(0-168.24)	0	0(0-72.72)	1	14.35(0.36-79.96)	1	5.88(0.15-32.77)
Other Urinary Organs	0	0(0-124.98)	0	0(0-155.44)	0	0(0-66.81)	0	0(0-51.83)	0	0(0-20.53)
Eye and Orbit	0	0(0-300.9)	0	0(0-369.08)	0	0(0-161.92)	0	0(0-126.51)	0	0(0-49.72)
Brain and Other Nervous System	0	0(0-6.6)	1	2.27(0.06-12.63)	1	1(0.03-5.56)	2	1.63(0.2-5.89)	4	1.24(0.34-3.17)

Endocrine System	1	6.56(0.17-36.57)	0	0(0-30.06)	0	0(0-12.99)	1	2.79(0.07-15.52)	2	2.18(0.26-7.87)
Thyroid	0	0(0-31.71)	0	0(0-39.25)	0	0(0-17.04)	1	3.64(0.09-20.3)	1	1.43(0.04-7.95)
Other Endocrine including Thymus	1	27.75(0.7-154.63)	0	0(0-128.4)	0	0(0-54.72)	0	0(0-43.6)	1	4.61(0.12-25.7)
Lymphoma	0	0(0-2.75)	0	0(0-3.52)	1	0.42(0.01-2.34)	5	1.68(0.54-3.91)	6	0.77(0.28-1.68)
Hodgkin Lymphoma	0	0(0-74.8)	0	0(0-96.39)	0	0(0-43.93)	0	0(0-36.28)	0	0(0-13.5)
Non-Hodgkin Lymphoma	0	0(0-2.86)	0	0(0-3.65)	1	0.44(0.01-2.43)	5	1.73(0.56-4.05)	6	0.8(0.29-1.75)
Myeloma	1	1.33(0.03-7.39)	4	6.84[p](1.86-17.5)	0	0(0-2.75)	1	0.59(0.01-3.28)	6	1.37(0.5-2.98)
Leukemia	0	0(0-2.71)	2	1.88(0.23-6.78)	2	0.82(0.1-2.97)	7	2.26(0.91-4.67)	11	1.38(0.69-2.47)
Lymphocytic Leukemia	0	0(0-9.94)	0	0(0-12.87)	0	0(0-5.64)	3	3.52(0.73-10.28)	3	1.39(0.29-4.05)
Acute Lymphocytic Leukemia	0	0(0-84.97)	0	0(0-107.31)	0	0(0-47.39)	1	10.11(0.26-56.34)	1	3.93(0.1-21.89)
Chronic Lymphocytic Leukemia	0	0(0-12.23)	0	0(0-15.89)	0	0(0-6.97)	2	2.9(0.35-10.48)	2	1.14(0.14-4.12)
Other Lymphocytic Leukemia	0	0(0-139.88)	0	0(0-182.95)	0	0(0-78.76)	0	0(0-57.27)	0	0(0-23.38)
Myeloid and Monocytic Leukemia	0	0(0-5.61)	1	1.92(0.05-10.71)	1	0.83(0.02-4.63)	2	1.28(0.16-4.64)	4	1.02(0.28-2.6)
Acute Myeloid Leukemia	0	0(0-6.91)	1	2.36(0.06-13.14)	0	0(0-3.77)	2	1.6(0.19-5.77)	3	0.94(0.19-2.75)
Acute Monocytic Leukemia	0	0(0-586.48)	0	0(0-799)	0	0(0-337.84)	0	0(0-268.89)	0	0(0-103.78)
Chronic Myeloid Leukemia	0	0(0-57.44)	0	0(0-74.22)	0	0(0-31.96)	0	0(0-24.08)	0	0(0-9.64)
Other Myeloid/Monocytic Leukemia	0	0(0-69.63)	0	0(0-87.89)	1	10.14(0.26-56.48)	0	0(0-26.63)	1	3.01(0.08-16.77)
Other Leukemia	0	0(0-11.01)	1	3.87(0.1-21.55)	1	1.74(0.04-9.68)	2	2.94(0.36-10.61)	4	2.16(0.59-5.54)
Other Acute Leukemia	0	0(0-30.92)	1	10.98(0.28-61.19)	0	0(0-18.68)	1	4.51(0.11-25.11)	2	3.18(0.38-11.47)
Aleukemic, Subleukemic and NOS	0	0(0-17.1)	0	0(0-22.02)	1	2.64(0.07-14.73)	1	2.18(0.06-12.14)	2	1.64(0.2-5.92)

Miscellaneous Malignant Cancer	15	5.77^{p}(3.23-9.52)	5	2.48(0.81-5.79)	14	3.08^{p}(1.68-5.16)	10	1.76(0.84-3.24)	44	2.96^{p}(2.15-3.98)

Table S36: Standardized-mortality ratios (SMRs) for cancer causes for non-cardia in all patients.
